# Primary cell culture systems to investigate host-pathogen interactions in bacterial respiratory tract infections of livestock

**DOI:** 10.3389/fcimb.2025.1565513

**Published:** 2025-05-09

**Authors:** Yenehiwot Berhanu Weldearegay, Louise Brogaard, Silke Rautenschlein, Jochen Meens, Peter Valentin-Weigand, Désirée Schaaf

**Affiliations:** ^1^ Institute for Microbiology, University of Veterinary Medicine Hannover, Foundation, Hannover, Germany; ^2^ Department of Biotechnology and Biomedicine, Section for Medical Biotechnology, Technical University of Denmark, Kgs. Lyngby, Denmark; ^3^ Clinic for Poultry, University of Veterinary Medicine Hannover, Foundation, Hannover, Germany

**Keywords:** nasal mucosa explants, tracheal organ cultures, precision-cut lung slices, air-liquid interface cultures, respiratory tract infections, organotypic models, livestock diseases

## Abstract

Respiratory infections of livestock represent a major health issue for the animals and cause high economic losses for the farmers. Still, little is known about the intricate interactions between host cells and the many different pathogens that cause respiratory diseases, leaving a substantial knowledge gap to be filled in order to develop effective therapies. Immortalized cell lines and two-dimensional cultures of primary respiratory epithelial cells do not reflect the complex architecture and functionality of the respiratory tract tissues. Thus, it is essential to develop and apply appropriate primary cell culture systems to study respiratory diseases. In human research, the use of complex cell culture systems, such as air-liquid interface (ALI) cultures, organoids and lung-on-chip, has proceeded significantly during the last years, whereas in veterinary research, these models are only rarely used. Nevertheless, there are several three-dimensional, primary cell culture systems available to study respiratory infections of livestock. Here, we give an overview on models that are currently used in this field: nasal mucosa explants, tracheal organ cultures, ALI cultures, and precision-cut lung slices. All these models align with the 3R principle, as they can replace animal experiments to some extent and the tissue material for these culture systems can be obtained from abattoirs or veterinary research facilities. We aim to encourage other researchers to use these versatile cell culture systems to drive investigations of respiratory tract infections of livestock forward. Finally, these models are not limited to infection research, but can also be applied in other research fields and can be transferred to other animal species than livestock.

## 1 Introduction

Respiratory infections of livestock cause major problems with regard to animal welfare and economic losses. In order to improve understanding of host-pathogen interactions, extensive research on pathogens, virulence factors, and the host’s immune response towards infection with respiratory pathogens is necessary. One of the most common methods to study host-pathogen interactions is the use of experimental animal models. However, this is ethically debatable mainly due to animal welfare, limited value for studying the early stages of disease, and, when using laboratory animals, the restricted transferability to the natural host species. Moreover, reproducibility of results, required time, and practicability make *in vivo* studies challenging (Steukers et al., 2011; Di Teodoro et al., 2018). Thus, suitable *in vitro* and *ex vivo* models, which resemble the *in vivo* situation and apply the 3R (replacement, reduction, and refinement) principle of Russel and Burch (Russell and Burch, 1959) are mandatory to study host-pathogen interactions.

Immortalized cell lines and two-dimensional (2D) cell culture models do not reflect the complexity of the respiratory tract and are therefore suboptimal for the study of host-pathogen interactions. Luckily, more reliable *in vitro* models of the respiratory tract have been developed and improved significantly during the last years, especially in human research due to the COVID-19 pandemic. Commonly used *in vitro* models in human research are primary human airway epithelial cells cultured under air-liquid interface (ALI) conditions, organoids, lung-on-chip, and precision-cut lung slices (PCLS) (Dichtl et al., 2024; Mahieu et al., 2024). However, the establishment and maintenance of organoids and lung-on-chip is quite challenging and expensive, and in veterinary science, these models are yet only rarely used.

Detailed reviews on complex human cell cultures systems have been published in the past (Cao et al., 2021; Viana et al., 2022) but to our knowledge, no reviews on primary cell culture systems from livestock are available. Thus, in this review, we present four different primary cell culture systems of the upper and lower respiratory tract that have been proven suitable for the study of host-pathogen interactions in respiratory infections of livestock – nasal mucosa explants (NME), tracheal organ cultures (TOC), ALI cultures, and PCLS. We focus on bacterial respiratory infections as there is currently a lack of studies on veterinary bacterial pathogens, and we want to encourage researchers to fill this knowledge gap in the future by using these *in vitro*/*ex vivo* models instead of animal experiments. For each model, we describe preparation and maintenance, possible applications, as well as limitations and future perspectives.

## 2 Nasal mucosa explants

NMEs for the study of diverse aspects of respiratory colonization and infection have been established from various livestock species, including pigs (Pol et al., 1991; Glazenburg et al., 1995; Glorieux et al., 2007; Van Poucke et al., 2010; Frydas et al., 2013; Tulinski et al., 2013; Starbæk et al., 2018), horses (Vandekerckhove et al., 2009; Glorieux et al., 2012; Vairo et al., 2013; Baghi and Nauwynck, 2014; Van Cleemput et al., 2019), cattle (Niesalla et al., 2009; Steukers et al., 2012; Yang et al., 2019), and sheep (Mazzetto et al., 2020; Zheng et al., 2023), but also humans (Read et al., 1995; Jackson et al., 1996; Jang et al., 2005; Wang et al., 2012; Cantero et al., 2013b; Gordhan et al., 2023). The nasal mucosa is the site of first contact for many pathogens and colonizing bacteria. Being able to study these pathogens using models that mimic this anatomical site is therefore of high importance, and the application of nasal mucosal tissue explant culture in the study of the initial interactions between host and pathogen thus have obvious benefits. NMEs are a 3R compliant *ex vivo* model as many NMEs can be obtained from one animal, thus attaining both replacement and reduction relative to applying *in vivo* experimentation. Moreover, in contrast to cell culture using immortalized or even primary nasal epithelial cells, NMEs carry the benefit of retaining the sophisticated three-dimensional (3D) structure of the nasal mucosal epithelium.

### 2.1 Preparation and maintenance

A guiding protocol for preparation and inoculation of NMEs can be found in **Supplementary Material 1**.

**Figure 1:**
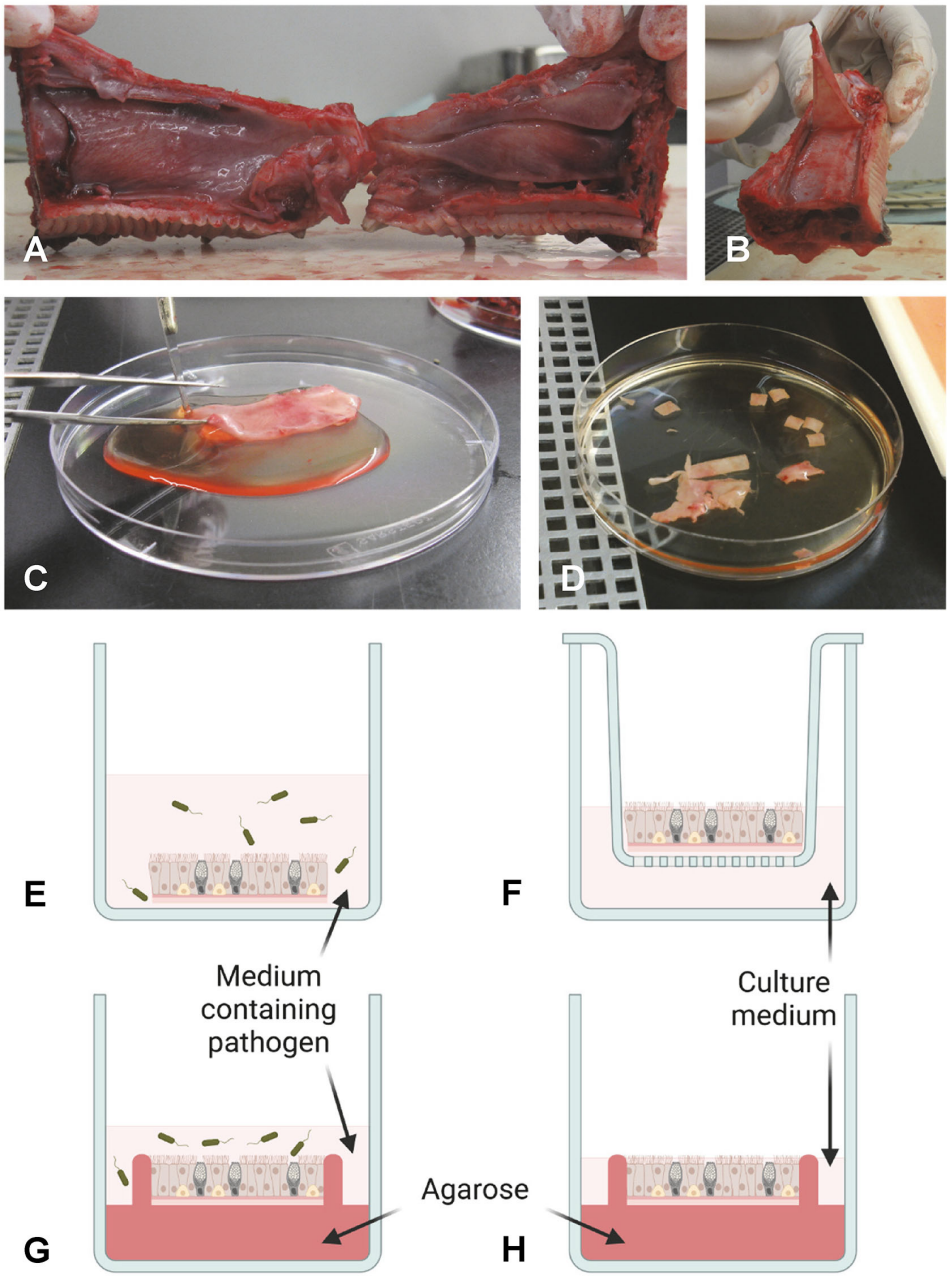
Schematic overview of preparation and culturing of NMEs. As an example, a pig snout is shown in **(A, B)**. To obtain NMEs, the interior of the nasal cavity is exposed **(A)** and the mucosal layer is stripped from the cartilage **(B, C)**. The tissue is then cut into appropriately sized pieces (NMEs) **(D)** and inoculated by submerging the NME in medium containing pathogen **(E)**. Following incubation and washing, the NME is placed on a scaffold (shown here as a transwell culture plate insert) and cultured at the air-liquid interphase **(F)**. Alternatively, a polarized setup can be employed. Prior to inoculation, the NME can be embedded in agarose to occlude the basolateral and side surfaces of the NME, thus allowing infection to occur only from the apical side **(G)**. Following incubation and washing, the embedded NME is cultured at the air-liquid interphase in fresh medium **(H)**. Drawings in panels G and H have been prepared taking inspiration from Frydas et al (Frydas et al., 2013). Photos provided by L. Brogaard, Department of Biotechnology and Biomedicine, Section for Medical Biotechnology, Technical University of Denmark, Kgs. Lyngby, Denmark. Created by L. Brogaard in BioRender. https://BioRender.com/dwhk64n.

The NME model is easily accessible to researchers, as it would usually not require any highly specialized lab equipment and can be set up in any standard cell culture lab. Tissue may be obtained at veterinary research facilities as the animals head will often be medical “waste” from experimental/educational surgeries, or it can be obtained from slaughterhouses. Euthanization of animals solely for the purpose of harvesting tissue for NMEs can thereby be avoided. NMEs are obtained by exposing the interior of the nasal cavity and carefully stripping the mucosal layer from the cartilage of *e.g.* the nasal septum and/or conchae (**Figures 1**). Tissue sheets are divided into samples of equal size, often approximately 2.5 cm² (Glazenburg et al., 1995), to be cultured individually. Depending on the species, age, and size, a fairly large number (often > 20) of NMEs may be obtained from one animal. The preparation of individual NMEs is usually followed by an acclimatization period of up to 24 hours. Exposure to pathogen(s) to start infection or colonization of NMEs is often achieved by incubating the tissue fully submerged in medium containing bacteria or virus for approx. 1–2 hours (**Figure 1**). Early studies including the use of NMEs have reported maintaining the tissue fully submerged in culture medium throughout the experiment (Pol et al., 1991; Glazenburg et al., 1995), but the more common practice is to place the NME apical side up on a scaffold and maintain it in an air-liquid interphase culture, mimicking *in vivo* conditions (**Figure 1**). Various types of scaffolds have been described, including fine-meshed metal gauze scaffolds (Glorieux et al., 2007; Oh et al., 2023; Stadejek et al., 2023), gelfoam blocks saturated with culture medium (Jang et al., 2005; Cantero et al., 2013b), cell-strainers adapted to serve as scaffold (Starbæk et al., 2018), transwell culture plate inserts (Zheng et al., 2023), and agarose plugs (Niesalla et al., 2009). It is often reported that a combination of RPMI-1640 medium and Dulbecco’s Modified Eagle Medium (DMEM) is used for culturing NMEs, usually supplemented with antibiotics when applying NMEs for the study of viral pathogens. For the study of bacterial pathogens, the acclimatization process may take place in the presence of antibiotics, but prior infection a thorough washing and an additional period of culturing in antibiotics-free medium are essential (Tulinski et al., 2013). Moreover, it has to be taken into account that animals often received antibiotics in the days prior to euthanization.

Importantly, the natural route of infection should be considered when applying the pathogen(s), *i.e.* either from the apical or the basolateral surface of the epithelium. To ensure access for the pathogen only to the apical side of NME during inoculation, polarized NME cultures have been established from pigs and horses, as well as humans (Read et al., 1995; Frydas et al., 2013; Vairo et al., 2013). To achieve this, the tissue is embedded in agarose, leaving only the epithelial surface exposed during inoculation, thus more closely mimicking the *in vivo* situation (**Figures 1E**).

Viability of the tissue is commonly assessed by confirming ciliary beating and checking for apoptotic cells by staining. Tissue integrity can be evaluated by monitoring tissue morphometrics, *e.g.* cell composition and epithelial thickness. These parameters indicate that the NME model is stable and viable in culture for up to 96 hours after isolation or even longer (Vandekerckhove et al., 2009; Van Poucke et al., 2010; Zhao et al., 2016).

### 2.2 Applications

Studies employing NMEs for investigation of viral pathogens far outnumber studies of bacterial pathogens. Studies on herpesviruses dominate equine and bovine NME literature, while porcine NMEs have been used for the study of influenza viruses, porcine reproductive and respiratory syndrome virus, African swine fever virus, as well as herpesvirus (pseudorabies virus). However, porcine as well as ovine NMEs have also been used for investigation of bacterial colonization. Porcine NMEs have been demonstrated to be a useful tool for investigation of *Staphylococcus aureus* colonization (Tulinski et al., 2013), and have been used to provide temporal resolution of *S. aureus* gene expression during the early phase of colonization and evaluate the effect of bacteriophage treatment on reduction of livestock-associated methicillin-resistant *S. aureus* colonization (Tulinski et al., 2014; Verstappen et al., 2016). In ovine NMEs, the antiviral effect of bacterial colonizers of the nasal mucosa has been investigated, suggesting that *Bacillus subtilis*-induced expression of antiviral factors contributed to restricting pseudorabies virus infection in the NME model (Zheng et al., 2023). The studies of bacteriophage treatment against *S. aureus* and antiviral effect of *B. subtilis* highlight the application of NMEs for evaluation of intervention strategies against pathogens that target the nasal mucosa, even though such studies are yet sparse. In addition to those already mentioned, just one other study investigating the antiviral effect of A-5021 [(10S,20R)-9-[[10,20-bis(hydroxymethyl)cycloprop-10-yl]methyl]guanine] on equine herpesvirus-1 in equine NMEs has utilized this potential of the model (Glorieux et al., 2012). Also highlighted by the study of the antiviral effect of *B. subtilis* in ovine NMEs is that NMEs may provide an excellent tool for investigating co-infections or interactions between colonizers and (opportunistic) pathogens. The nasal mucosal microbiota is a complex and diverse community, and any infection by an inhaled or opportunistic pathogen would always occur in the presence of colonizers that may potentially exacerbate or inhibit the infection. The fact that NMEs can be colonized by relevant bacteria *in vitro* promotes their application in this context. However, this possibility has so far not been extensively explored in livestock NMEs, whereas human NMEs have been utilized to investigate the effect of influenza B virus on the association of *Neisseria meningitidis* with the nasal mucosa and to demonstrate the role of herpes simplex virus type 1 in facilitating *S. aureus* invasion of the nasal mucosa (Read et al., 1999; Wang et al., 2012).

### 2.3 Limitations and future perspectives

Inherently, the NME model will comprise tissue resident immune cells, such as dendritic cells and macrophages, as well as mucus-secreting goblet cells, which can be significant for the model’s ability to accurately mimic the conditions encountered by a pathogen or commensal colonizer during first and early contact with the host’s nasal mucosa. However, it has to be considered that recruitment of circulating lymphocytes to the site of infection does obviously not occur which limits its use for studying immune responses during later stages of infection.

Most studies employing NMEs have been focused on characterizing aspects of the pathogen under investigation, *e.g.* replication dynamics, infectivity, tissue and cell tropism, and pathogen gene expression dynamics during the infection. Some studies have also utilized the NME model to investigate the host response during infection. Human NMEs have been successfully used to characterize the innate immune response to *S. aureus* biofilm formation, showing that the bacterium induces a predominantly pro-inflammatory state in the nasal mucosa early in the process (Cantero et al., 2013a, Cantero et al., 2015). Various aspects of the host response during viral infections have also been investigated using livestock-derived NMEs, *e.g.* the migration of tissue resident immune cells in equine NMEs during equine herpesvirus-1 infection and the potential relevance for systemic viral spread (Baghi and Nauwynck, 2014; Zhao et al., 2017). Porcine NMEs have been used to characterize host antiviral gene expression during influenza A virus infection in a study that, in part, investigated the ability of the NME model to accurately mimic the *in vivo* antiviral innate immune response. Moreover, this study also evaluated technical aspects such as inter-experiment variation and importantly the effect on host transcription of keeping the nasal mucosal tissue in culture for several days (Starbæk et al., 2018). The authors concluded that the process of harvesting and culturing the tissue had significant impact on expression of genes related to *e.g.* inflammation and apoptosis, even in the absence of viral infection. This is important when using the NME model for investigation of pathogen or host gene expressions, as the culturing process itself may influence the results. Nonetheless, the NME model has been proved a valuable tool in facilitating sophisticated investigation of *e.g.*, viral replication and host cell tropism, viral-bacterial co-/super-infection, effect of biofilm on host nasal mucosa, mapping host and pathogen transcriptional landscapes during colonization or infection, and efficiency of antiviral drug candidates in inhibition of viral infection and migration. The NME model does however seem somewhat under-utilized for investigation of bacterial infection and colonization, but it has potential to become a relevant tool for this purpose in the future.

## 3 Tracheal organ cultures

TOC have been described in the literature from human (Dolin and Smith, 1975) and various animal species, including laboratory animals (Wolterbeek et al., 1996; Kishimoto et al., 2021), domestic animals (Leeming et al., 2006) as well as from livestock (Thomas et al., 1987; DeBey and Ross, 1994; Niang et al., 1998). Notably, the majority of these publications is about TOC from chicken and other birds. This could be due to the fact that suitable culture conditions for air-liquid interface (ALI) cultures of the bird’s respiratory tract have not been published so far and only few studies describe the use of precision-cut lung slices (PCLS) of immature chicken embryos to investigate pathogen-host interactions (Abd El Rahman et al., 2010; Bryson et al., 2020). Thus, in poultry research, TOC represent an alternative model to investigate the avian upper respiratory tract in the context of infections with especially viruses, bacteria, but also parasites and have been used for over 50 years (Colwell and Lukert, 1969; Abdul-Wahab et al., 1996; Zhang et al., 2012; Knab et al., 2020). TOC were also applied in the context of pharmacological investigations addressing decongestants and mucolytic agents or mycotoxins (Cardeilhac et al., 1972; Dudley and Cherry, 1977). TOC can be considered a highly reproducible *ex vivo* model with a cell composition closely mimicking the *in vivo* situation in birds and other animal species. However, in this review, we will focus on the preparation and application of TOC in poultry research as literature on other primary cell culture models in this research area is sparse.

### 3.1 Preparation and maintenance

**Figure 2:**
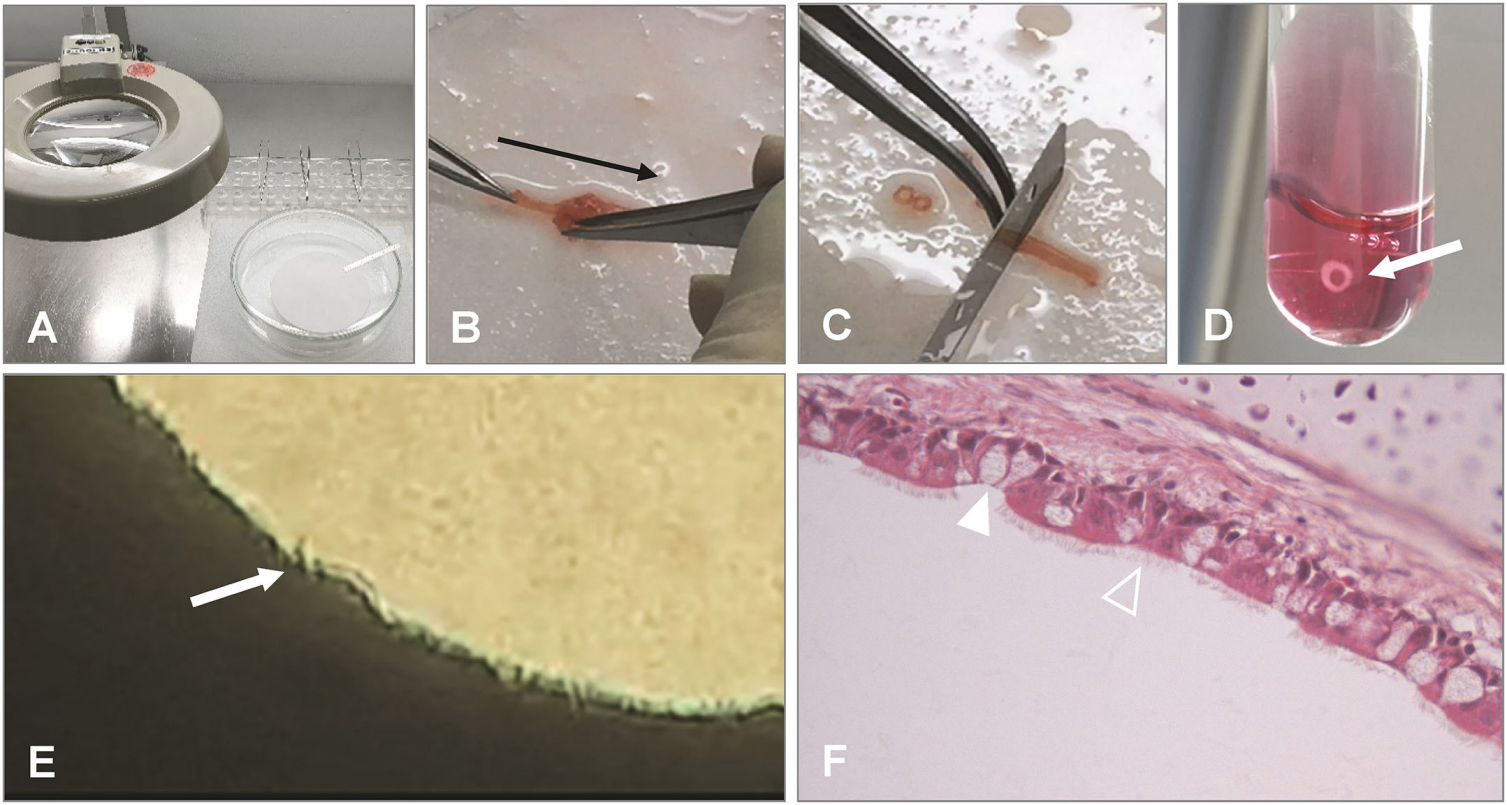
Preparation of chicken TOC. **(A)** Preparation should be done under a magnification glass in a petri dish with pre-warmed medium and on filter paper. **(B)** After removal of the trachea from the embryo or post hatch bird, connective tissue is stripped off (black arrow indicates direction of stripping). **(C)** Cutting of the trachea into 0.8–1 mm evenly thick rings with a sharp blade manually or with a tissue chopper. **(D)** Placement of the ring in pre-warmed medium (arrow indicates TOC) and incubation in an overhead rotator at 37°C. **(E)** Microscopical evaluation of the tracheal ring after incubation for ciliary activity using an inverted microscope (arrow indicates cilia and a clear lumen of the ring without mucus accumulation). **(F)** Histological section of the TOC showing goblet cells (filled arrowhead) and epithelial cells with cilia (open arrowhead). Photos provided by N. Rüger and S. Rautenschlein, Clinic for Poultry, University of Veterinary Medicine Hannover.

TOC can be prepared from embryos or post-hatched birds (de Wit et al., 2013; Dowgier and Bickerton, 2020), the latter playing an important role in the evaluation of vaccine efficacy against especially infectious bronchitis virus infection in chickens. TOC have been prepared from various bird species (Colwell et al., 1973; Petersen et al., 2012) and species-specific but also age-related differences may be observed in the duration and intensity of ciliary activity. Pheasant TOC have shown ciliary activity up to 70 days, and TOC derived from chicken embryos maintained ciliary activity longer compared to day-old chicks (Gerganov and Surtmadzhiev, 1982a). For the collection of the trachea, embryos at about 1–2 days before hatch or post-hatch birds are humanely sacrificed. The trachea is immediately carefully removed and placed in pre-warmed medium (for example Medium 199 with Hank’s salts supplemented with antibiotics). Subsequently, the trachea will be placed in pre-warmed medium in a petri dish on sterile filter paper (**Figures 2**). *Ex vivo* the trachea will be stripped off connective tissue (**Figure 2**), and subsequently rings of about 1 mm thickness will be cut manually with a sharp microtome blade or with a tissue chopper (**Figure 2**) (Hennion, 2015). The rings are placed in pre-warmed medium. Single rings can be incubated in 5 ml sterile plastic tubes with 1 ml medium (**Figure 2**), and incubated at 37-38°C in an overhead rotating rack at the lowest rotating speed or in roller cell culture bottles to prevent accumulation of mucus within the lumen of the ring (Jones and Hennion, 2008; Hennion, 2015), which prolongs survival of the rings compared to static cultures. After about four days, when stress related (innate immune) responses of the TOC due to the preparation process have waned (Reemers et al., 2009), infection studies can be initiated. Importantly, it has to be considered that even from embryos the tracheae may not be fully sterile, although bacteria may not be detected by classical microbiological procedures, microflora may be identified by Illumina sequencing (Taylor et al., 2020; Ding et al., 2022; Faldynova et al., 2024). Therefore, some effects of this microflora on the structural and non-structural cells including innate immune reactions of the TOC cannot be ruled out. Tissue can remain viable at least four weeks, or, depending on the age or species the TOC were collected from, shorter or longer (Cherry and Taylor-Robinson, 1970). Viability can be confirmed by ciliary activity, which is investigated by using an inverted microscope. For evaluation of ciliary beats, the ring is divided into 10% sections. The ciliary activity is documented for each section, which adds up to total ciliary activity. About 100% ciliary activity is expected for intact TOC, while it wanes in infected or dying tissue until total ciliostasis with or without loss of cilia may be observed. Depending on the experimental protocol, the media may have to be changed when cultures are maintained for an extended period.

For a detailed protocol, the reader is referred to the publication by Hennion et al. (Hennion, 2015).

### 3.2 Application

TOC have traditionally been used for virus propagation and titration (Cook et al., 1976; Dowgier and Bickerton, 2020), but they are also applicable for the investigation of host-pathogen interactions. Ciliary activity is an important read-out parameter to assess the impact of an infection on the tissue viability. Thus, only rings with full ciliary activity should be used for experimental infections. Histopathological examination of the TOC can be performed to identify pathogen-induced lesion development including loss of cilia and epithelial cells, edema of the epithelial layer or changes in the number of goblet cells (**Figure 2**). Pathogens may be identified by (microbiological) culture, molecular methods (including quantitative PCR (qPCR), *in situ* hybridization) or by antigen staining using specific peroxidase- or fluorescence-labeled antibodies. TOC structures involved in the pathogen-host-interaction may be visualized by antibodies, lectins, or specific staining procedures with for example Alcian blue for mucus (Winter et al., 2008; Zhong et al., 2024). By using confocal microscopy, pathogen and host structures can be detected simultaneously. Besides the detection of the pathogen by electron microscopy or molecular methods, also the expression of immune related genes including Toll-like receptors, interaction of pathogens with respective ligands, and immune responses can be investigated by reverse transcription quantitative real-time PCR (RT-qPCR) or microarray systems (Reemers et al., 2009; Barjesteh et al., 2016). Enzyme-linked immunosorbent assays (ELISA) or bioactivity assays for the detection of soluble factors released by the TOC into the supernatant are rarely used, as most systems are less sensitive than molecular methods, and the individual TOC is very small that only low levels of molecules of interest may be found in the medium.

### 3.3 Limitations and future perspectives

The most significant limitation in investigations of host-pathogen-interaction in TOC is the lack of the specific immune response. Although residual numbers of T and B cells may be found at the beginning of the culture of TOC, these cells have a short survival time without the addition of specific growth factors (Kothlow et al., 2008; Linti et al., 2024). However, TOC provide an excellent model for host-pathogen investigations of not only birds in the order galliformes such as turkeys, chicken or quail but have also been applied for columbiformes and anseriformes of different species (Colwell et al., 1973; Kocan et al., 1978; Gerganov and Surtmadzhiev, 1982b; Abdul-Wahab et al., 1996; Petersen et al., 2012). We expect that TOC may also be used for other bird species in the future. TOC can be used for the investigation of innate immune responses, which has been shown after mono- and co-infection with various pathogens (Chhabra et al., 2016; Sid et al., 2016; Ruger et al., 2021), and for the comparison of strains of pathogens (Raj and Jones, 1996). The use of TOC allows a statistically meaningful number of replicates and an easy comparison of genotypes and bird species with respect to questions related to pathogenesis under well controlled and unified conditions (Petersen et al., 2012; Hartmann et al., 2015), which would be difficult under *in vivo* conditions. Though, variation between experiments using different birds or embryonated eggs from different parents cannot be fully excluded due to genetic variations of the donor animals and variable microbiota colonizing the respiratory tract of the donor. An additional limitation is the cellular complexity of the TOC, which may not allow pinning down the cell type associated with for example expressed cytokines/chemokines in tissue homogenates. Therefore, single cell RNA sequencing technologies are expected to be used increasingly for the analysis of complex tissues including TOC.

## 4 Primary respiratory epithelial cells cultured under air-liquid interface conditions

It is commonly accepted that primary cells derived from the host species are in general more reliable than immortalized cell lines, which differ genetically and phenotypically from the original host cell (Alge et al., 2006; Pan et al., 2009). However, the 2D submerged culture of primary respiratory epithelial cells does not reflect the sophisticated architecture and the expression profile of the native respiratory epithelium (Kaartinen et al., 1993; Pezzulo et al., 2011). Moreover, these cultures lack the most important defense mechanism of the respiratory tract, the mucociliary clearance mechanism (Ostrowski and Nettesheim, 1995; Gray et al., 1996). In contrast, primary respiratory epithelial cells cultured under ALI conditions develop a well-differentiated pseudostratified epithelium, characterized by the formation of an epithelial barrier and by the presence of ciliated, mucus producing (goblet cells), and basal cells, thereby mimicking the *in vivo* airway epithelium very closely (Whitcutt et al., 1988). Despite some differences regarding the cell population and the immune competence of ALI cultures compared to the *in vivo* respiratory epithelium, the general gene expression and (patho-) physiological reactions are similar (Ross et al., 2007; Dvorak et al., 2011; Pezzulo et al., 2011; Mathis et al., 2013).

ALI cultures with respiratory epithelial cells have been used frequently for various applications in different fields of respiratory research, *e.g.*, toxicology, pharmacokinetics, pathology, virology, and bacteriology. In contrast to human ALI cultures, respiratory epithelial cells from livestock are usually readily available at low costs from slaughterhouses. Nevertheless, the reproducibility of primary cells is low due to a high interindividual variability (Abraham et al., 2011; Stewart et al., 2012). Moreover, the development of a well-differentiated respiratory epithelium *in vitro* is a complex process, comprising the initial attachment, proliferation, and polarization of the cells, thickening of the cell layer and mucociliary differentiation, as well as dedifferentiation, cell death and remodeling of the cell layer (Gray et al., 1996), which makes the establishment and maintenance of ALI cultures quite challenging.

For a comprehensive characterization of caprine, ovine, and bovine ALI cultures the reader is referred to recent respective publications (O’Boyle et al., 2017; Cozens et al., 2018a, Cozens et al., 2018b; O’Boyle et al., 2018; Strassle et al., 2021). This review aims to give an overview of the preparation and maintenance as well as possible applications of ALI cultures from livestock.

### 4.1 Preparation and maintenance

The general procedure of ALI culture preparation and maintenance is similar for all host species. However, there are some species-specific differences in the medium supplementation or culture conditions, which will be highlighted in the following sections. A detailed protocol for the preparation of ALI cultures with primary porcine respiratory epithelial cells can be found in **Supplementary Material 2**.

**Figure 3:**
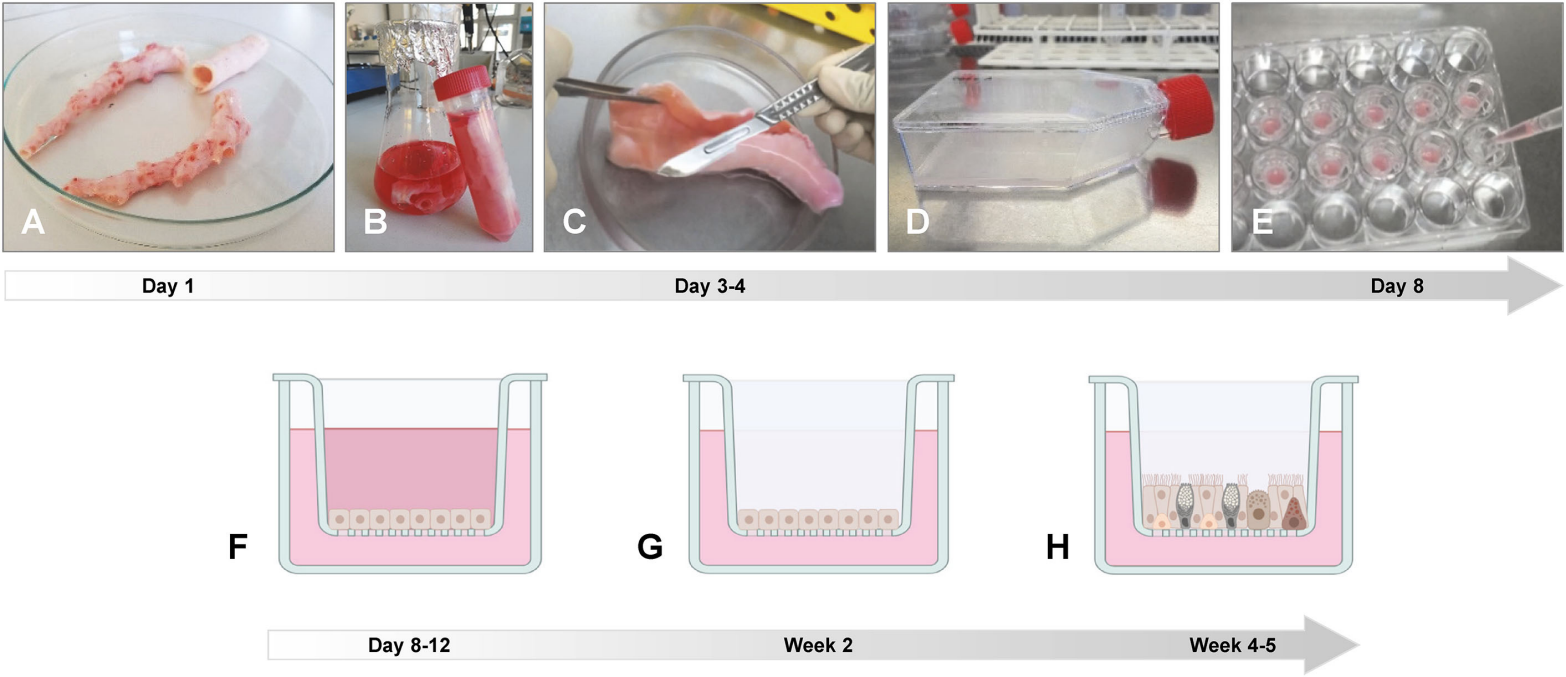
Preparation of ALI cultures from porcine trachea/bronchi. **(A)** Lungs from slaughtered animals from the abattoir are transported on ice to the laboratory and the trachea and/or the main bronchi are separated from the connective lung tissue. **(B)** The trachea and/or the main bronchi are incubated in digestion buffer at 4°C for up to three days. **(C)** Epithelial cells are scraped off from the luminal surface using a scalpel blade and transferred to DMEM supplemented with 10% FBS. After filtration and washing, cells are incubated in non-coated plastic cell culture dishes at 37°C for 1–2 h to reduce fibroblast contamination. **(D)** Non-adherent epithelial cells are then transferred to a collagen I-coated cell culture flask and incubated at 37°C until cells are confluent. **(E)** Epithelial cells are detached from the cell culture flask and seeded on collagen IV-coated polycarbonate membranes with a pore size of 0.4 µm and **(F)** incubated under submerged conditions for four days. **(G)** Medium from the apical compartment is removed and **(H)** the epithelial cells differentiate under ALI conditions within 3–4 weeks. Photos provided by **(D)** Schaaf, Institute for Microbiology, University of Veterinary Medicine Hannover, Germany. Created by D. Schaaf in BioRender. https://BioRender.com/b62a774.

The lungs from livestock can be easily and cost-effectively obtained from abattoirs. After removing the connective tissue (**Figure 3**), trachea/bronchi are transferred to a “digestion buffer” - medium (*e.g*., DMEM) supplemented with protease, DNase, antibiotics, and antimycotics - and incubated for up to 72 hours (h) at 4°C (**Figure 3**). The supplements and the incubation time differ between the species and/or the research groups (**Supplementary Table 1**). Another possibility to dissociate the cells is the incubation in 0.25% trypsin-0.6 mM EDTA at 37°C for 2 h (Abraham et al., 2011). Afterwards, the cells are harvested either by centrifugation (Radi and Ackermann, 2004), by rinsing the tissue pieces (Oslund et al., 2010), by agitation (Mao et al., 2009), or by scraping the cells from the tissue with a scalpel blade (**Figure 3**) (Schwab et al., 2010). To remove cell detritus and mucosal residues, it is recommended to wash the cell suspension and to filter it through a cell strainer (40-70 µm) (Schwab et al., 2010). Fibroblast contamination can be reduced by incubation of the cell suspension in non-coated plastic cell culture dishes at 37°C for 1–2 h, as fibroblasts rapidly adhere to plastics (Mao et al., 2009). The non-adherent epithelial cells can then be transferred to “submerged growth medium” (SGM; **Supplementary Table 2**) to propagate them in collagen-coated tissue culture flasks (**Figure 3**) or to transfer them directly onto collagen-coated permeable membrane inserts (**Supplementary Table 3**) in a transwell system consisting of two chambers (apical and basal; **Figure 3**). The epithelial cells are kept under submerged conditions until confluence (approximately 2–7 days; **Figure 3**) and are then introduced to ALI conditions. For this, the medium is removed from the apical chamber (“air”) and “ALI medium” (**Supplementary Table 4**) is added to the basal chamber (“liquid”; **Figure 3**). At the interface of air and liquid, the respiratory epithelial cells can build a well-differentiated pseudostratified epithelium, consisting of ciliated, mucus producing, and basal cells, within an average of three weeks (**Figure 3**). During differentiation, the medium in the basal chamber is changed every 2–3 days and the cells should be washed at least once per week with neutral buffer solution (*e.g.*, PBS, HBSS) to maintain the homeostatic balance of mucus and cells. Usually, the fully differentiated epithelium remains stable for additional three weeks, allowing long-term experiments (O’Boyle et al., 2017; Cozens et al., 2018b). Notably, Strässle et al. described caprine ALI cultures that were stable for up to two months – afterwards, the ciliary activity decreased and cells died (Strassle et al., 2021).

The composition of the SGM is essential for the growth of respiratory epithelial cells (Mao et al., 2009) and can be decisive for the success of cell differentiation under ALI conditions (O’Boyle et al., 2018). The presence of serum in the SGM promotes the proliferation of respiratory epithelial cells (Mao et al., 2009) and seems to be important for the subsequent differentiation under ALI conditions (Abraham et al., 2011). However, serum is also reported to induce squamous differentiation of respiratory epithelial cells (Lechner et al., 1984), indicating that the concentration of serum or serum substituent (*e.g*., Ultroser G) in the “ALI medium” has to be adjusted very carefully. According to our literature research, the optimum serum concentration in SGM is 5-10% fetal bovine serum (FBS) or 0.5 mg/ml bovine serum albumin (BSA; **Supplementary Table 2**) and in “ALI medium” 0.5 mg/ml BSA or 2% Ultroser G (**Supplementary Table 4**).

Proliferation and differentiation of respiratory epithelial cells is a complex process, which requires the precise supplementation of growth factors and hormones in the “ALI medium”, and even though some studies used commercially available media successfully (Wang et al., 2020; Genna et al., 2023; Muller et al., 2023), they sometimes fail to support differentiation (Luengen et al., 2020). Epidermal growth factor, retinoic acid, and triiodo-L-thyronine are all components that should be carefully adjusted in the “ALI medium” to ensure that they optimally support proliferation and differentiation without causing any adverse effects (Gray et al., 1996; Yoon et al., 1997; Cozens et al., 2018a; O’Boyle et al., 2018).

In addition to growth factors and hormones, the physical properties of the membrane insert, such as material (*e.g.*, PC, PET, PTFE), pore size, pore density, and coating of the membrane might affect the differentiation of respiratory epithelial cells (Davenport and Nettesheim, 1996; Widdicombe et al., 2003; Lee et al., 2011; Cozens et al., 2018a; O’Boyle et al., 2018). A high pore-density, allowing the sufficient availability of nutrients across the membrane, was required for optimal differentiation of bovine bronchial and ovine tracheal epithelial cells (Cozens et al., 2018a; O’Boyle et al., 2018). However, the pore-density of the membrane insert is often not indicated by the manufacturer.

Oxygen tension is supposed to influence proliferation and differentiation of respiratory epithelial cells, as it is reported for neural and mesenchymal stem cells (Chen et al., 2007; Panchision, 2009; Liu et al., 2015). Cozens et al. and O’Boyle et al. tested different oxygen concentrations (7%, 14%, and 21%) and found that optimal ciliation and proliferation of bovine bronchial and ovine tracheal epithelial cells occurred at 14% oxygen (Cozens et al., 2018a; O’Boyle et al., 2018).

Even though passaged primary respiratory epithelial cells retain their ability to differentiate into ciliated and mucus-producing cells, they differ from freshly isolated cells, as they completely dedifferentiate under submerged conditions resulting in a homogenous cell population. Passaged cells have been shown to establish cell-cell-connections more efficiently (Abraham et al., 2011) but are delayed in the development of ciliated cells compared to freshly isolated cells (Karp et al., 2002; Lee et al., 2005; Wiszniewski et al., 2006).

Finally, a common challenge with primary cell culture systems is the high variability between the donor individuals (Abraham et al., 2011; Stewart et al., 2012; Russell et al., 2018; Luengen et al., 2020), which can be only addressed by an appropriate number of biological and technical replicates. However, this variability reflects the differences between individuals among the same species occurring also *in vivo*, indicating that this culture system is mimicking the *in vivo* situation reliably.

### 4.2 Applications

This section describes a selection of possible read-out methods and applications for ALI cultures with respiratory epithelial cells from livestock. In contrast to human ALI cultures, literature about livestock ALI cultures is limited. However, methods used for human ALI cultures may also be transferred to ALI cultures from livestock.

**Figure 4:**
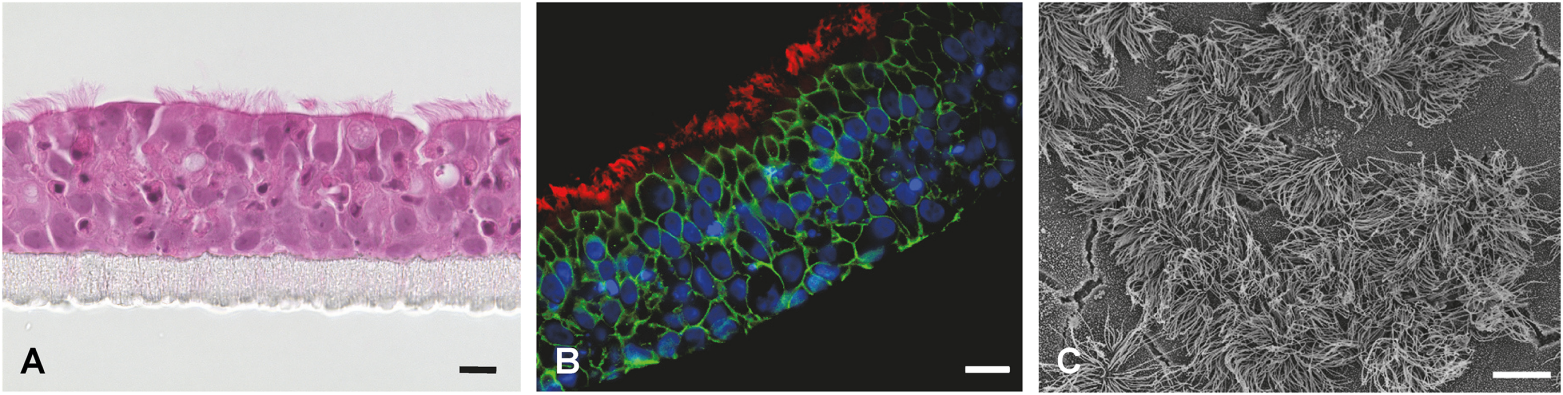
Fully differentiated primary porcine bronchial epithelial cells (PBEC) under ALI conditions. **(A)** Hematoxylin-eosin staining of PBEC (D. Schaaf, Institute for Microbiology, University of Veterinary Medicine Hannover, Germany). **(B)** Immunofluorescence staining of PBEC under ALI conditions. Visualization of cilia (β-tubulin, red), tight junctions (β-catenin, green), and nuclei (DAPI, blue) (D. Schaaf, Institute for Microbiology, University of Veterinary Medicine Hannover, Germany). **(C)** SEM of PBEC (M. Rohde, Helmholtz Center for Infection Research, Braunschweig, Germany). Bars represent 10 µm.

Measurement of the trans-epithelial electrical resistance (TEER) is the most common method to evaluate the epithelial barrier integrity of the respiratory epithelial cell layer. Regardless of the species origin, TEER usually peaks during the first days under ALI conditions, then decreases and stays stable for the remainder of the culturing (Lam et al., 2011; Bateman et al., 2013; Meng et al., 2016; O’Boyle et al., 2017; Cozens et al., 2018b; Wang et al., 2018). Barrier functionality can also be assessed by the measurement of a dextran flux across the cell layer (Kreft et al., 2015; Ramezanpour et al., 2016; Su et al., 2020) or immunofluorescence staining of tight or adherens junctions (**Figure 4**; **Supplementary Table 5**). Furthermore, immunofluorescence staining or immunohistochemistry allow the visualization of ciliated cells, mucus producing cells, and basal cells (**Supplementary Table 5**). Hematoxylin-eosin staining of cross sections indicates the cellular composition, thickness of the barrier, and the extent of ciliation (**Figure 4**) (Abraham et al., 2011; Bateman et al., 2013; O’Boyle et al., 2017; Cozens et al., 2018b; Wang et al., 2018; Strassle et al., 2021), whereas the periodic acid-Schiff (PAS) reaction colors the mucus (Oslund et al., 2010; Schwab et al., 2010; Abraham et al., 2011; O’Boyle et al., 2017). When using transparent membrane inserts, cell layers as well as cilia can be monitored by conventional light microscopy (Schwab et al., 2010; Abraham et al., 2011; Wang et al., 2018; Strassle et al., 2021). Transmission electron microscopy (TEM) (Mao et al., 2009; Khoufache et al., 2010; Oslund et al., 2010; Schwab et al., 2010; Ma et al., 2016; O’Boyle et al., 2017; Cozens et al., 2018b; Wang et al., 2020) as well as scanning electron microscopy (SEM; **Figure 4**) (Mao et al., 2009; Abraham et al., 2011; Lam et al., 2011; Xue et al., 2015; Meng et al., 2016; O’Boyle et al., 2017; Cozens et al., 2018b; Wang et al., 2018; Strassle et al., 2021; Genna et al., 2023) allows the ultrastructural visualization of ciliated and goblet cells. Additionally, the presence of mucus producing cells may also be estimated by the expression of mucins by RT-qPCR (Oslund et al., 2010; Schwab et al., 2010).

The lactate dehydrogenase (LDH) release assay is a commercial assay commonly used to determine the damage induced by a pathogen or a substance and can be easily applied to ALI cultures (Li et al., 2016; Meng et al., 2016; Su et al., 2020; Qin et al., 2023). In order to examine the immunomodulatory or inflammatory response of the host cells, the activation of inflammatory pathways as well as the induction of cytokines/chemokines can be analyzed by RT-qPCR (Khoufache et al., 2010; Quintana et al., 2011; Xue et al., 2015) or Western blotting (Ma et al., 2016). In addition, cytokine and/or chemokine levels in the supernatant can be determined using ELISA (Jiang et al., 2017; Wang et al., 2018).

For bacterial infection studies, it is feasible to determine the bacterial replication rate, *e.g.*, by plating of supernatant and cell lysates (Meng et al., 2016), by qPCR (Wang et al., 2020), or by color-changing units (Strassle et al., 2021), and to visualize attachment of the pathogen to respiratory epithelial cells via immunofluorescence staining (Meng et al., 2019).

ALI cultures with primary respiratory epithelial cells from livestock can be applied to study the biology of the airway epithelium and respiratory tract diseases (Oslund et al., 2010; Quintana et al., 2011; Abugisisa et al., 2023), to investigate interactions between host cells and viral (Goris et al., 2009; Bateman et al., 2013; Kirchhoff et al., 2014; Wu et al., 2016; Van Cleemput et al., 2017; Yang et al., 2019; Peng et al., 2020; Shin et al., 2020; Gultom et al., 2021; Peng et al., 2021; Muller et al., 2023), bacterial (Xue et al., 2015; Li et al., 2016; Ma et al., 2016; Meng et al., 2016; Jiang et al., 2017; Su et al., 2020; Wang et al., 2020; Strassle et al., 2021; Qin et al., 2023), or fungal (Khoufache et al., 2010) pathogens, to study co-infections of the respiratory tract (Meng et al., 2019), or they might be used for drug transport studies (Lin et al., 2007; Min et al., 2016) or toxicological risk assessment (Friesen et al., 2022). Moreover, ALI cultures from livestock, especially from swine, can provide a suitable model for the human respiratory tract (Lam et al., 2011; Genna et al., 2023).

Finally, ALI cultures with primary respiratory epithelial cells from livestock offer numerous possibilities of applications and read-out methods, a lot more than mentioned above, and are therefore promising *in vitro* models to study the biology of the respiratory epithelium and respiratory diseases.

### 4.3 Limitations and future perspectives

Most ALI cultures consist only of epithelial cells, which does not reflect the *in vivo* respiratory tract appropriately, as it also comprises immune cells, fibroblasts, chondrocytes, endothelial cells, etc. To address this aspect, co-culture systems (*e.g.*, respiratory epithelial cells and fibroblasts) have been developed (Albers et al., 2015; Abs et al., 2019). These studies showed that co-cultivation with fibroblasts improves the differentiation of porcine tracheal and equine bronchial epithelial cells. Co-culture systems including fibroblasts and/or immune cells are also reported for human ALI cultures (Harrington et al., 2014; Kletting et al., 2018; De Rudder et al., 2020; Friesen et al., 2022). Leach et al. developed a 3D airway ‘organ tissue equivalent’ by including pulmonary fibroblasts, solubilized lung extracellular matrix, and hydrogel substrate with tunable stiffness and porosity (Leach et al., 2023). Another factor, which can affect the epithelial development and the immune response but is only rarely addressed in *in vitro* systems, is the respiratory microbiota (De Rudder et al., 2020). Furthermore, most ALI cultures from livestock consist of epithelial cells from the conducting airways (mainly trachea and bronchi), whereas we found only one article about bovine alveolar epithelial cells, co-cultured with bovine pulmonary arterial endothelial cells (Lee and Chambers, 2019). Since the epithelium of gas-exchanging airways differs from that of the conducting airways, it might be interesting to include also ALI cultures consisting of alveolar epithelial cells in future studies.

Remarkably, to our knowledge no reports about ALI cultures with respiratory epithelial cells from poultry exist. Although it was shown that primary avian tracheal epithelial cells develop to ciliated cells, goblet cells, and basal cells under submerged conditions (Shen et al., 2010), they fail to build a pseudostratified epithelium comparable to the native avian respiratory tract. Regarding the occurrence of respiratory pathogens (*e.g.*, avian influenza virus, *Mycoplasma gallisepticum*) leading to increased mortality in poultry, it might be worthwhile to establish the ALI culture system for avian respiratory epithelial cells to investigate host-pathogen interactions *in vitro*.

Overall, ALI cultures with respiratory epithelial cells from livestock represent a major area of development and a promising cell culture system for future studies in the field of respiratory research.

## 5 Precision-cut lung slices

PCLS are 3D *ex vivo* organ models, which closely mimic the *in vivo* situation and have significant impact and value in translational science (Alsafadi et al., 2020). Since first described by Placke and Fisher in 1987 (Placke and Fisher, 1987), PCLS have been established from lungs of different animal species including humans during the last decades. PCLS have advantages over cell culture as they reflect the structural and functional heterogeneity of lung tissue. The microarchitecture of lung tissue including vessels, airways, and nerves, which are all embedded in the lung parenchyma, is intact and enables investigations under physiological conditions (Martin et al., 1996; Schleputz et al., 2011). In addition, the morphological organization of the cellular components and their response to stimuli are very close to the *in vivo* situation in the lung (Hays et al., 2003; Harrigan et al., 2004; Henjakovic et al., 2008). PCLS are composed of various cell types, which can be classified as structural (such as epithelial, endothelial, lymphatic, smooth muscle, and fibroblastic cells) and non-structural cell types, mainly cells of the immune system (like macrophages, neutrophils, dendritic cells, T cells, and B cells) (Lyons-Cohen et al., 2017; Niehof et al., 2017). Additional advantages of PCLS include the ease of preparation, low costs, good reproducibility, and the stability of cells *ex vivo* (Ruigrok et al., 2019; Weldearegay et al., 2019; Ram-Mohan et al., 2020). Moreover, PCLS serve as an efficient and rapid screening method to identify suitable experimental animal models, thereby reducing the necessity for large-scale animal usage (Gerhards et al., 2021).

During our literature research, we found that PCLS from livestock have been used in different research areas, *e.g.*, lung physiology and pathophysiology, pharmacology and gene therapy, as well as respiratory infection research. Whereas several studies on viral respiratory pathogens have been published during the last twenty years, only few publications on bacterial respiratory infections of livestock can be found (Weldearegay et al., 2019; Qin et al., 2021; Votsch et al., 2021).

Recently, there have been several comprehensive reviews on PCLS with regard to human lung biology and disease modelling as well as the investigation of therapeutic targets (Liu et al., 2019; Alsafadi et al., 2020; Viana et al., 2022; Lam et al., 2023). Thus, in this review we focus on PCLS as a powerful tool to investigate respiratory bacterial infections of livestock.

### 5.1 Preparation and maintenance

A detailed protocol for the preparation of porcine PCLS can be found in **Supplementary Material 3**.

**Figure 5:**
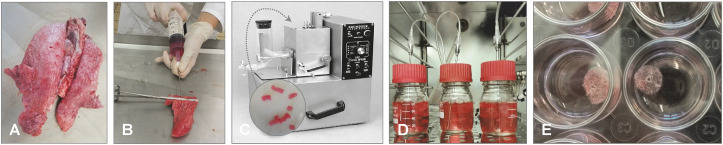
Preparation of porcine PCLS. **(A)** Lungs from slaughtered animals from the abattoir are transported on ice to the laboratory. **(B)** The cranial lobes of apparently healthy lungs are dissected and filled with 37°C low melting-point agarose through a cannula introduced in the bronchus. Filled lung lobes are kept on ice until the agarose becomes solid. **(C)** By using a tissue coring tool, cylindrical pieces of lung tissue with a bronchioles in the middle are punched out and cut into approximately 300 µm thin tissue slices by using a tissue slicer or vibratome (here: Krumdieck tissue slicer, model MD 4000-01; TSE Systems, Chesterfield, MO, USA). **(D)** The tissue slices are transferred to cell culture medium (*e.g.*, RPMI) supplemented with antibiotics and antimycotics and bubbled with a normoxic gas mixture at 37°C to remove the agarose from the airway lumen. **(E)** PCLS can be maintained for up to two weeks in cell culture medium (*e.g.*, RPMI) supplemented with antibiotics and antimycotics in, *e.g.*, a 24-well plate. Photos provided by D. Schaaf, Institute for Microbiology, University of Veterinary Medicine Hannover, Germany.

PCLS are prepared following standard procedures, which implement the use of low-melting agarose prepared with buffer or cell culture medium (*e.g.*, RPMI 1640 medium) (Placke and Fisher, 1987). The concentration of agarose varies from 0.4% to 3% - lower concentrations should be used for small and fragile lobes, whereas higher percentages can be used for lungs from humans and bigger animals (Alsafadi et al., 2020). Lungs from livestock can be easily obtained from a local slaughterhouse. The cranial and accessory lobes (big/adult animals) or the whole lung (small/young animals) can be used, respectively. The lung lobes/lungs are filled slowly with warm (37°C) low-melting agarose via a cannula introduced in the (main) bronchus/trachea, until all the air spaces are filled with agarose (**Figure 5**). These lobes are then kept on ice or in chilled buffer until the agarose inside the tissue solidifies. Once the agarose becomes solid, thin tissue slices (150-500 µm) can be prepared using a tissue slicer or vibratome (**Figure 5**). Optional, before slicing, cylindrical tissue sections can be punched out of the solidified tissue by using a tissue-coring tool to prepare round tissue slices that fit into well plates. The agarose is removed from the airways by washing, *e.g.*, on a shaker or by bubbling with a normoxic gas mixture (Paddenberg et al., 2014) (**Figure 5**). Finally, the slices are maintained in cell culture medium (*e.g.*, RPMI 1640 medium), supplemented with antibiotics and antimycotics to eliminate contaminating bacteria and fungi, and can remain viable for up to two weeks (Neuhaus et al., 2017; Temann et al., 2017; Weldearegay et al., 2019) or even ≥ 40 days (chicken) (Bryson et al., 2020). Medium should be changed every day to minimize the effect of endogenously released mediators (Lambermont et al., 2014) as well as to remove any residual agarose. Depending on the pathogen used for the experimental infection, the medium should be changed to a medium without antibiotics/antimycotics, at least one day before infection.

### 5.2 Applications

Ciliary activity is the major means of controlling tissue vitality using light microscopy (Votsch et al., 2021). However, to quantify the impact of a pathogen or other agents on the ciliary motility, the ciliary beat frequency has to be determined by high-speed video microscopy, as described by Dresdner and Wong in 1985 (Dresdner and Wong, 1985). Viability may also be assessed using metabolic tests (*e.g*., WST-1 or MTS assay) (Neuhaus et al., 2017; Weldearegay et al., 2019) and cytotoxicity assays (*e.g*., LDH-release assay) (Remot et al., 2021; Votsch et al., 2021; Gaudino et al., 2023; Qin et al., 2023), as well as live-dead staining (Neuhaus et al., 2017; Gaudino et al., 2023) and flow cytometry (Gaudino et al., 2023).

To quantify bronchoconstriction following infection or drug administration, the bronchial cavity areas can be measured by light microscopy (Qin et al., 2021) or tissue traction microscopy can be applied to measure contractile forces of airway smooth muscles (Ram-Mohan et al., 2020).

**Figure 6:**
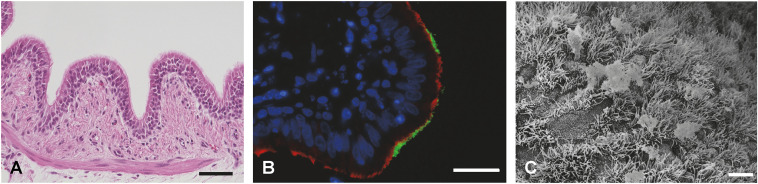
Visualization of PCLS. **(A)** Hematoxylin-eosin staining of porcine PCLS; bar represents 50 µm (R. Spriewald, Institute for Microbiology, University of Veterinary Medicine Hannover, Germany). **(B)** Immunofluorescence staining of porcine PCLS infected with *Bordetella bronchiseptica*. Bacteria are shown in green, cilia (β-tubulin) in red, and nuclei (DAPI) in blue; bar represents 20 µm (D. Schaaf, Institute for Microbiology, University of Veterinary Medicine Hannover, Germany). **(C)** SEM of porcine PCLS; bar represents 5 µm (M. Rohde, Helmholtz Center for Infection Research, Braunschweig, Germany).

Histopathological examinations give important information on the overall tissue structure and alterations due to infection or treatment with other agents (Weldearegay et al., 2019; Qin et al., 2021; Votsch et al., 2021) (**Figure 6**). In contrast, immunohistochemistry or immunofluorescence staining allows the identification and visualization of specific tissue structures (*e.g.*, cilia, adherence junctions) or pathogens, thereby enabling the localization of the pathogen on the tissue slice as well as the documentation of host cell-pathogen interactions (Meng et al., 2015; Weldearegay et al., 2019; Remot et al., 2021; Votsch et al., 2021; Qin et al., 2023) (**Figure 6**). Ultrastructural visualization of tissue structures and pathogens is possible with SEM (Votsch et al., 2021) (**Figure 6**) or TEM (Weldearegay et al., 2019; Gaudino et al., 2023).

Even though emerging analysis methods, such as mass spectrometry and next generation sequencing, have so far mainly focused on human PCLS, this demonstrates their applicability across species for RNA-sequencing (Huang et al., 2019; Stegmayr et al., 2021; Crue et al., 2023) as well as for proteomic and metabolomics analyses (Mossina et al., 2017; Khan et al., 2021). These methods would significantly advance the study of host-pathogen interactions in respiratory tract infections of livestock.

Transcriptomic studies using microarray analysis and PCLS from different species revealed a number of interesting results, which could be utilized in identifying multifactorial mode of drug action in the treatment of infectious diseases (Reamon-Buettner et al., 2019) as well as a better understanding of innate immune responses towards infectious agents (Weldearegay et al., 2023; Li et al., 2024). Furthermore, the host’s immune response upon infection can be evaluated by cytokine expression profiles and the identification of specific immune pathways using RT-qPCR (Remot et al., 2021; Gaudino et al., 2023; Weldearegay et al., 2023) or by detection of cytokines in the supernatant using ELISA, Luminex xMAP^®^ Technology, or Western blotting (Dresen et al., 2021; Remot et al., 2021; Gaudino et al., 2023; Qin et al., 2023; Weldearegay et al., 2023).

The number of colonizing bacteria can either be determined by plating of supernatants and tissue lysates on agar plates (Meng et al., 2015; Remot et al., 2021; Votsch et al., 2021; Qin et al., 2023), by (duplex) qPCR (Weldearegay et al., 2019; Hanske et al., 2023), or by RT-qPCR (Gaudino et al., 2023). Moreover, the presence of virulence (-associated) factors during infection of PCLS can be analyzed by Western blotting (Dresen et al., 2021; Votsch et al., 2021) or RT-qPCR (Weldearegay et al., 2023).

The methods described here represent only a small selection of what is possible with this versatile *ex vivo* model, as methods described for human PCLS can likewise be applied to PCLS from animals.

### 5.3 Limitations and future perspectives

Although PCLS provide important information on host-pathogen interactions, one limitation of PCLS is that the slices, including the airways, are submerged in a liquid medium, which does not represent the *in vivo* situation, where the airways and alveoli are largely filled with air. In pharmacological studies, the route of drug administration remains to be a challenge since slices are submerged in medium containing the drug/compound of interest. This complicates assessment of the effect of administration routes, *e.g.* systemic delivery or local application *in vivo* (Liu et al., 2019). Similarly, regarding respiratory infections, the natural (*i.e.*, nasal) route of infection cannot be mimicked with this model.

Another important limitation of the PCLS model is the lack of immune cell recruitment and adaptive immunity. Even though resident immune cells responsible for host defense mechanisms are present, their activation and release of cytokines may not initiate subsequent differentiation and recruitment of other immune cells towards the site of infection, which limits the extent of immune response using PCLS (Liu et al., 2019; Viana et al., 2022). However, the early innate immune response can be studied in PCLS and alterations in the inflammatory response upon infection with (bacterial) pathogens have been reported in different studies (Remot et al., 2021; Gaudino et al., 2023; Qin et al., 2023; Weldearegay et al., 2023).

The viability of cells in PCLS being on average restricted to two weeks (Neuhaus et al., 2017; Temann et al., 2017; Weldearegay et al., 2019) limits the application of PCLS for chronic infection and toxicity studies (Viana et al., 2022). In order to address this, Bailey and colleagues used poly(ethylene glycol)-based hydrogel platforms to embed the PCLS and successfully prolonged the life span of viable PCLS (Bailey et al., 2020).

For more efficient utilization of PCLS, some research groups tested different methods of preservation such as cold or hypothermic storage and cryopreservation and found that structural and functional stability including immune functions were to a great extent maintained (Rosner et al., 2014; Bai et al., 2016; Watson et al., 2016; Tigges et al., 2021; Patel et al., 2023; Melo-Narvaez et al., 2025).

Despite these limitations, PCLS provide a useful tool to study host cell-pathogen interactions as well as the early pulmonary immune response during mono- and co-infection of the respiratory tract in livestock.

## 6 Conclusion and outlook

In the present review, we summarized preparation, maintenance, and applications of four different primary cell culture systems of the upper and lower respiratory tract in the context of respiratory infections in livestock. While especially PCLS and ALI cultures are widely used in human health research, the use of the four models described here in veterinary research is still scarce.

The main advantages of these models are listed in **Table 1**. The nearly unlimited access to the starting material (organs and tissues from slaughtered animals), simplifies the examination of adequate numbers of biological and technical replicates. In addition, early steps in host-pathogen interaction can be studied with high temporal resolution, which is difficult to achieve in *in vivo* experimental infections due to the high demand of laboratory animals in this type of experiments. The tissue based systems are derived from the upper (NME, TOC) and lower (PCLS) respiratory tract, which allows the comparison of region-specific epithelial cell type reactions.

**Table 1:** Overview of the benefits and limitations of the presented primary cell culture systems.

	NME	TOC	PCLS	ALI
**Benefits**	Can be applied to various animal species			
	Mimic *in vivo* situation very closely			
	Raw material can be easily obtained at low costs			
	Many technical replicates from one animal			
	Apply to the 3R principle (*i.e.*, replacement of animal experiments)			
	Wide range of possible applications			
	Temporal resolution of early infection events possible			
	Retains structure of original tissue			Defined cell composition
	Tissue resident immune cells present			
	Readily available after short acclimatization process			
	No special equipment required	Observation of ciliary beating via light microscopy		Analysis of epithelial differentiation and barrier function
**Limitations**	Limited life span of only a few weeks			
	No recruitment of circulating immune cells			
	Inter-experiment variation due to differences between donor individuals			
	Submerged cultivation does not reflect *in vivo* situation			Establishment and maintenance challenging
	Limited possibility of genetic manipulation			Production takes several weeks and is expensive

Existing limitations (**Table 1**), like the limited life span, the lack of immune cell recruitment and adaptive immunity, and limited genetic manipulation methods are subjects of ongoing and future improvements, *e.g.*, by transfection of PCLS or epithelial cells growing under ALI conditions.

In summary, the four primary cell culture systems offer the potential for relatively high throughput examination of specific aspects of host-pathogen interaction in respiratory mono- and co-infections. They apply to the 3R principle (replacement), thereby reducing ethical implications compared to *in vivo* studies and can bridge the gap between classical 2D *in vitro* models and experimental animal models.

Future applications are not restricted to classical respiratory infections in livestock, but also zoonotic pathogens like *Streptococcus suis* or even the characterisation of organs from genetically multi-modified pigs in the field of xenotransplantation might be interesting.
