# Supplementary material for: Primary cell culture systems to investigate host-pathogen interactions in bacterial respiratory tract infections of livestock
Source: Front Cell Infect Microbiol. 2025 May 9;15:1565513. doi: 10.3389/fcimb.2025.1565513 (PMC12098631; doi:10.3389/fcimb.2025.1565513)
Supplement: Supplementary file 1 [file SupplementaryFile1.pdf]

frontiers

Supplementary Material

1 General protocol for preparation of (porcine) nasal mucosal explants (NMEs)

Provided by Louise Brogaard (Department of Biotechnology and Biomedicine, Section for Medical Biotechnology, Technical University of Denmark, Kgs. Lyngby, Denmark)

1.1 Important Notes

This protocol should be considered a guideline, and specific adjustments according to user needs should be implemented, including any changes appertaining to the animal species of interest, as the following protocol is prepared using the pig as an example. Photos provided by Louise Brogaard (photos 1-8) and Malene Rask Thoftgaard (photos 9-10).

Prior to establishing NMEs: Consider treating animals with antibiotics prior to euthanasia.

1.2 Preparation of Media

Growth medium:

	Company	Cat. No.	Final concentration
DMEM	Thermo Fisher Scientific	61965026	50%
RPMI 1640	Thermo Fisher Scientific	72400021	50%
Penicillin/Streptomycin	Thermo Fisher Scientific	15140122	100 U/ml, 0.1 mg/ml
Gentamicin	Thermo Fisher Scientific	15710049	0.1 mg/ml

DMEM: Dulbeccos Modified Eagle Medium

Transport medium:

	Company	Cat. No.	Final concentration
PBS (with calcium/magnesium)	Thermo Fisher Scientific	14040133	
Penicillin/Streptomycin	Thermo Fisher Scientific	15140122	1000 U/ml, 1 mg/ml
Gentamicin	Thermo Fisher Scientific	15710049	0.5 mg/ml
Amphotericin B	Thermo Fisher Scientific	15290018	5 µg/ml

PBS: Phosphate-buffered saline

Washing medium:

	Company	Cat. No.	Final concentration
PBS (with calcium/magnesium)	Thermo Fisher Scientific	14040133	
Penicillin/Streptomycin	Thermo Fisher Scientific	15140122	100 U/ml, 0.1 mg/ml

All media should be 37°C at use.

1.3 Procedure

1. The euthanized pig is exsanguinated, and to make it easier to subsequently remove the snout, the head is first removed from the body. Using a saw, the snout is removed by a vertical cut from right in front of the eyes to the oral commissure. The remaining work is carried out in a laminar flow hood. Skin and muscle tissue is stripped from the snout. The interior of the snout is regularly moistened with pre-warmed transport medium.

2. The nasal mucosa is exposed by making an incision with a scalpel in the palate approx. 2 mm from the septum (photo 1) and underlying cartilage/bone is cut with surgical cutting pliers (photo 2). Take care not to touch the surface of the mucosa. A parallel cut is made on the top of the snout. When both cuts are complete, the snout can be broken open (photo 3). Take care not to tear the mucosa. Make sure to regularly moisten the mucosa with pre-warmed transport medium.

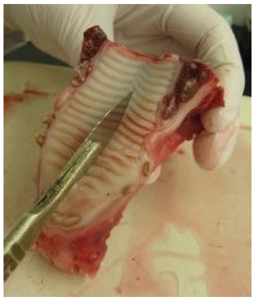
Photo 1

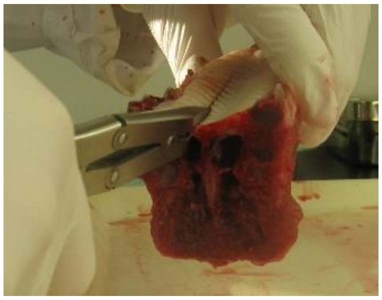
Photo 2

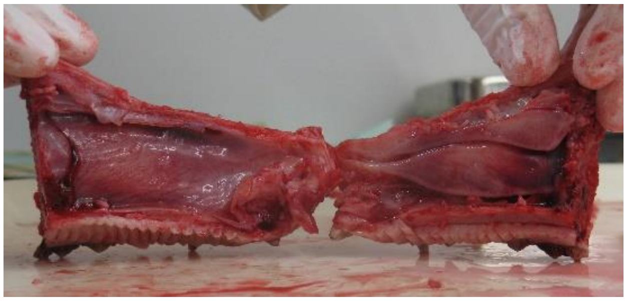
Photo 3

3. **Stripping mucosa from septum:** with a scalpel, make a cut along the edge of the piece of mucosa to be removed (yellow dots, left, photo 4). Grab the mucosal layer by the edge (where the snout was sawed off) and gently peel the mucosal layer off; adhesions to the underlying cartilage are carefully and gently broken with a sharp scalpel – no back and forth cutting motion, gentle pressure with scalpel is sufficient (photo 5). When freed from the cartilage, place the mucosa apical side up in pre-warmed transport medium in a petri dish (photos 6, 7).

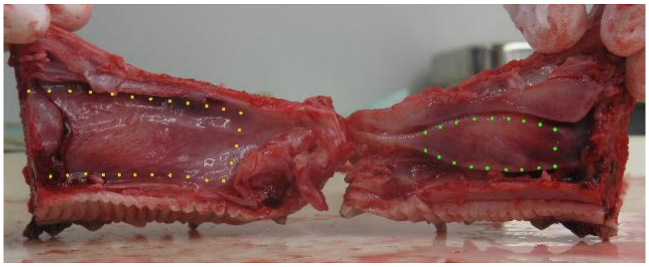
Photo 4

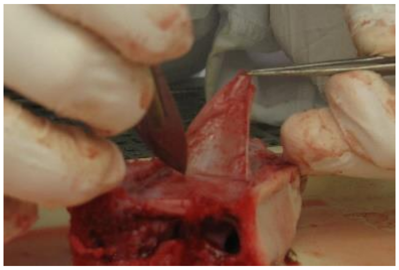
Photo 5

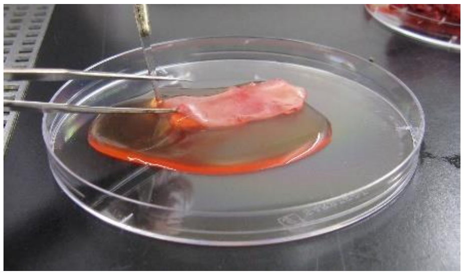
Photo 6

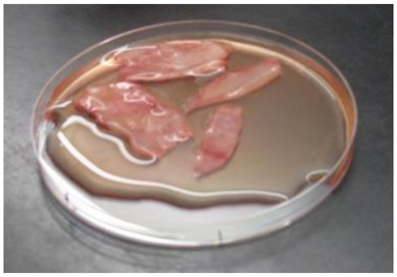
Photo 7

4. **Stripping mucosa from conchae:** with a scalpel, make a cut along the edge of the piece of mucosa to be removed (green dots, right, photo 4). Grab the mucosal layer by an edge and gently peel the mucosal layer off. If necessary, adhesions to the underlying cartilage can be carefully and gently broken with a sharp scalpel, but this will likely not be necessary as mucosa from the conchae peels of easily compared to mucosa from the septum. When freed from the cartilage, place the mucosa apical side up in pre-warmed transport medium in a petri dish (photo 7).

5. **Dividing into NMEs:** the edges of each sheet of mucosa may have sustained mechanical damage during recovery, so it may be advantageous to trim the outer 0.5 mm off each sheet before continuing. All cuts to the mucosa are performed with a <u>sharp</u> scalpel (change the blade as often as needed) by applying firm downward pressure – <u>no back and forth cutting motion.</u> The trimmed mucosa is then divided into appropriately sized squares (photo 8).

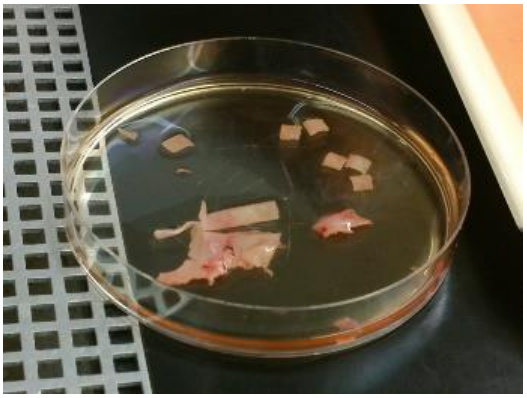

Photo 8

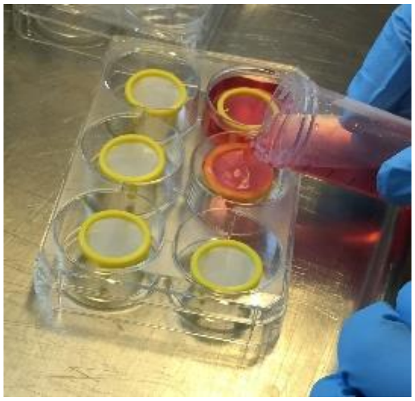

Photo 9

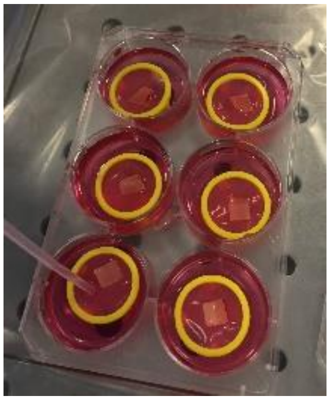

Photo 10

6. **Culturing of NMEs:** culturing of NMEs can be carried out in 6-weel plates using modified cell strainers (photo 9), here exemplified with 100 µm pore size cell strainers (Corning, Cat. No. 431752) with the top plastic edge and mesh sides cut off and placed upside down in the well. Each well is filled with pre-warmed growth medium (photo 9) so that the NMEs can be placed exactly at the air-liquid interphase (photo 10). Acclimatization can be done by culturing for up to 24 hours at 37°C at 5% CO_2. Viability can be assessed continuously by checking for ciliary beating.

7. **Maintenance and inoculation of NMEs:** after acclimatization, NMEs can be inoculated with the pathogen of interest. NMEs are moved from the air-liquid interphase and placed in a 24-well plate, one NME per well. Using 0.5 ml pre-warmed washing medium, each NME is washed twice. Each NME is then immersed in medium containing the pathogen for an appropriate amount of time, *e.g.*, one hour. After inoculation, the NMEs are again washed twice with 0.5 ml pre-warmed washing medium, placed back at the air-liquid interphase, resupplied with fresh pre-warmed growth medium, and cultured at 37°C at 5% CO_2. It may be appropriate to change the growth medium at regular intervals, *e.g.*, every 24 hours.
