# Supplementary material for: Primary cell culture systems to investigate host-pathogen interactions in bacterial respiratory tract infections of livestock
Source: Front Cell Infect Microbiol. 2025 May 9;15:1565513. doi: 10.3389/fcimb.2025.1565513 (PMC12098631; doi:10.3389/fcimb.2025.1565513)
Supplement: Supplementary file 2 [file SupplementaryFile2.pdf]

Supplementary Material

1 General protocol for preparation of air-liquid interface (ALI) cultures with primary (porcine) respiratory epithelial cells

Provided by Désirée Schaaf (Institute for Microbiology, University of Veterinary Medicine Hannover, Germany)

1.1 Important Notes

This protocol should be considered a guideline, and specific adjustments according to user needs should be implemented, including any changes appertaining to the animal species of interest, as the following protocol is prepared using the pig as an example. Photos provided by Désirée Schaaf.

1.2 Preparation of Media

Wash medium:

	**Company**	**Cat. No.**	**Final concentration**
DMEM	Thermo Fisher Scientific	41966052	
Penicillin/Streptomycin	Merck	P4333	100 U, 0.1 mg/ml
Gentamicin	Carl Roth	HN09.2	50 µg/ml
Amphotericin B	Merck	A2942	2.5 µg/ml

Incubation medium:

	**Company**	**Cat. No.**	**Final concentration**
DMEM	Thermo Fisher Scientific	41966052	
Penicillin/Streptomycin	Merck	P4333	100 U, 0.1 mg/ml
Gentamicin	Carl Roth	HN09.2	50 µg/ml
Amphotericin B	Merck	A2942	2.5 µg/ml
Protease	Merck	P5147	1 mg/ml
DNase	Merck	11284932001	10 µg/ml

Airway Epithelial Cell Growth Medium (AEGM):

	**Company**	**Cat. No.**	**Final concentration**
Airway Epithelial Cell Basal Medium	PromoCell	C-21260	
Airway Epithelial Cell Growth Medium SupplementMix	PromoCell	C-39165	
Retinoic acid	Merck	R2625	15 ng/ml
Y-27632 Dihydrochloride	Tocris	1254	10 µM
Fetal bovine serum (heat-inactivated)	Bio&SELL	FBS.S.0615	10%
Penicillin/Streptomycin	Merck	P4333	100 U, 0.1 mg/ml
Gentamicin	Carl Roth	HN09.2	50 µg/ml
Amphotericin B	Merck	A2942	2.5 µg/ml

ALI medium:

	Company	Cat. No.	Final concentration
Airway Epithelial Cell Basal Medium	PromoCell	C-21260	50%
DMEM	Thermo Fisher Scientific	41966052	50%
Airway Epithelial Cell Growth Medium SupplementMix	PromoCell	C-39165	
Retinoic acid	Merck	R2625	15 ng/ml
Bovine serum albumin	Merck	A7638	0.5 mg/ml
Penicillin/Streptomycin	Merck	P4333	100 U, 0.1 mg/ml

1.3 Procedure

Day 1:

1. Transport lungs from freshly slaughtered pigs on ice to the laboratory.
2. Separate the trachea and/or the main bronchi from the lung tissue using a scalpel or butcher's knife, put them in a beaker filled with cold phosphate-buffered saline (PBS).
   Note: The remaining steps should be carried out under a laminar flow hood and under sterile conditions.
3. The adherent tissue is thoroughly removed from the trachea/bronchi using microscopic scissors (Photo 1).
4. Trachea and bronchi are incubated in **wash medium** at 4°C for 24 hours (h; Photo 2).

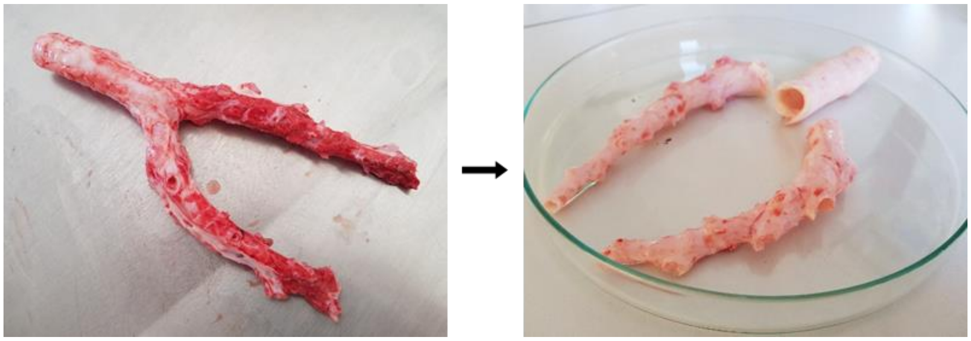

Photo 1

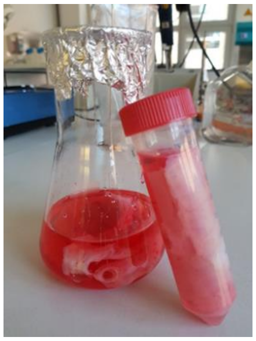

Photo 2

Day 2:

5. Remove wash medium and incubate trachea/bronchi in **incubation medium** at 4°C for 24 h (bronchi) or 48 h (trachea), respectively.

Day 3:

6. Open trachea/bronchi longitudinal and harvest tracheal/bronchial epithelial cells by gently scraping on the luminal surface with a scalpel blade (Photo 3).

7. Transfer cells to a 50 ml tube filled with DMEM (Thermo Fisher Scientific, Cat. No. 41966052) supplemented with 10% fetal bovine serum (FBS; Bio&SELL, Cat. No. FBS.S.0615; Photo 4, arrow indicates cell pellet).
8. Centrifuge cells (250 × *g*, 10 min) and wash once with PBS.
9. Resuspend cell pellet in 10 ml **Airway Epithelial Cell Growth Medium (AEGM)** and filter cell suspension using a cell strainer (100 µm pore size; Photo 5) and add 40 ml AEGM.
   Note: Pool cells from at least three different animals to reduce inter-individual variation (30 ml cell suspension in total + 120 ml AEGM).

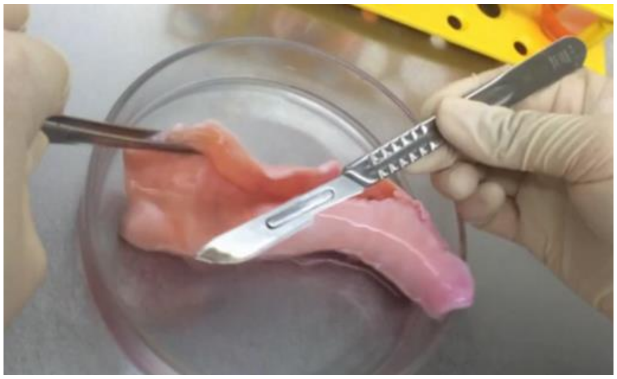
Photo 3

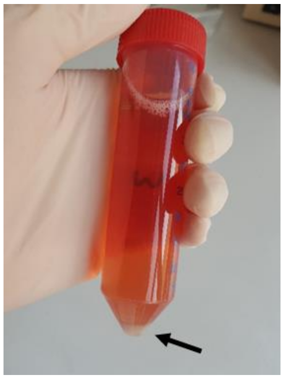
Photo 4

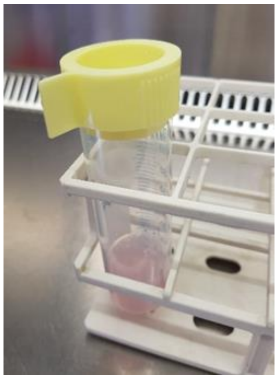
Photo 5

10. Transfer cell suspension to large non-coated cell culture dishes (50 ml/dish; Sarstedt, Cat. No. 83.3903) and incubate at 37°C and 5% CO_2 for 1-2 h to reduce fibroblast contamination (Photo 6).
    Note: Fibroblasts adhere rapidly to non-coated plastic surfaces while epithelial cells need several hours.
11. Check cell viability under the light microscope (Photo 7).
    Note: Viable cells show high ciliary activity indicated by rapid movement of the cells.

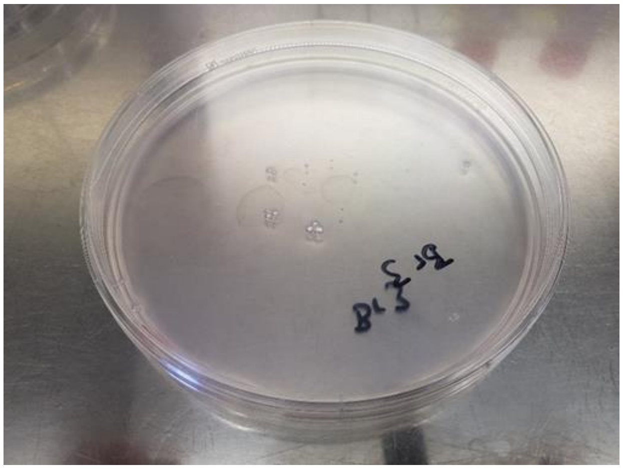
Photo 6

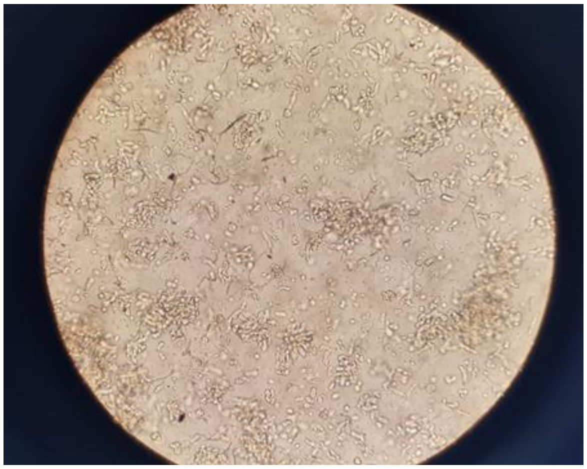
Photo 7

12. Collect cells in 50 ml tubes and separate them by pipetting up and down.
    Note: The cells are clumped together during incubation and clumped cells will not adhere properly to the cell culture flasks.

13. Transfer the cell suspension to collagen I (Merck, Cat. No. C3867; final concentration 10 µg/cm²)-coated T75-cell culture flasks (15 ml/flask) and incubate at 37°C and 5% CO_2 for 24 h (Photo 8).
    Note 1: Do not move the flasks too much to prevent the cells from clumping together again.
    Note 2: Cell suspension from three different animals (i.e., from three trachea or six main bronchi, 150 ml in total) can be divided into 10 cell culture flasks.

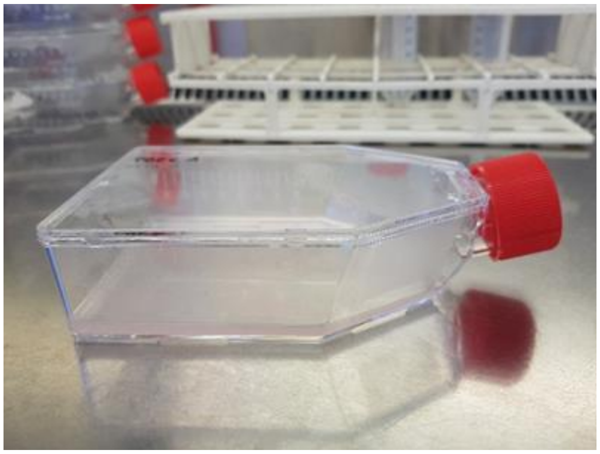

Photo 8

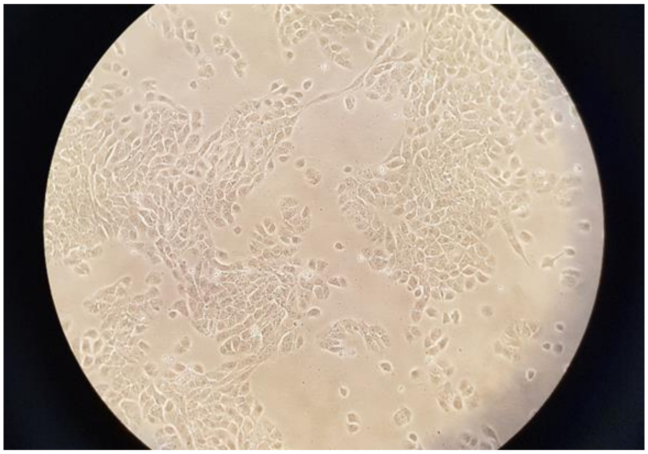

Photo 9

Day 4:

14. Wash cells twice with PBS to remove non-adherent epithelial cells, add fresh AEGM, and incubate cells further at 37°C and 5% CO_2 until they build a confluent cell layer. Check cell confluence by using a light microscope (Photo 9, approximately 60% confluence).
15. Change medium every 2-3 days if necessary.

Day 8:

16. When cells reach 80-90% confluence, they can be dissociated and seeded on transwell filters or prepared for freezing.
17. Dissociate cells using 0.5% trypsin-EDTA (3 ml/flask; Thermo Fisher Scientific, Cat.-No. 15400054) and incubate for 15-20 min at 37°C until cells are completely detached, add AEGM to stop enzyme activity (7 ml/flask) and centrifuge cells (250 × *g*, 10 min).
18. Wash cells once with PBS.
19. Resuspend cell pellet in 5 ml **AEGM** and determine number of viable cells (*e.g.*, by staining with 0.1% erythrosine B).
    Note: For cryo-preservation, resuspend the cell pellet in AEGM + 30% FCS + 10% dimethyl sulfoxide (DMSO; Merck, Cat. No. D2650) at a density of 5×10^6 cells/vial (800 µl/vial). Freeze cells in an insulated container at -80°C overnight. For long-term storage, transfer vials to liquid nitrogen. One vial of cells is sufficient for 1-2 T75-cell culture flasks.
20. Adjust cell number to 1×10^6 cells/ml and seed cells on collagen IV-coated porous supports (0.4 µm pore size, diameter 6.5 mm; VWR, Cat. No. 734-2742; Photo 10).
    → 7.5×10^5 cells/cm² (2.5×10^5 cells per 6.5 mm support = 250 µl/support)
21. Add 500 µl **AEGM** to the basal compartment and incubate at 37°C and 5% CO_2 for 24 h.

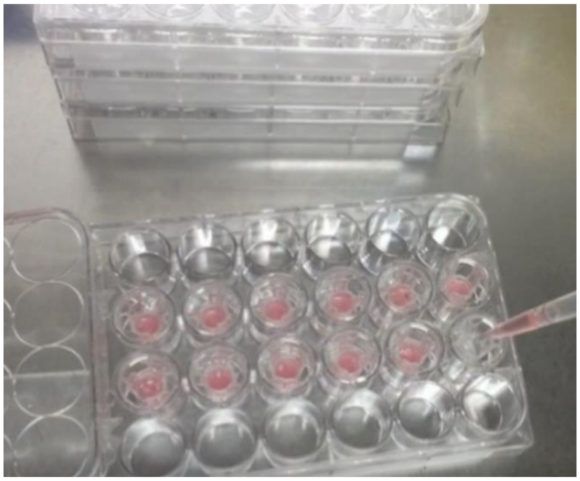

Photo 10

Day 9:

22. Wash cells twice with Hank's Balanced Salt Solution with Ca^{2+}/Mg^{2+} (HBSS+) on a horizontal shaker for 5 min.
23. Transepithelial electrical resistance (TEER) can be assessed using a voltohmmeter (*e.g.*, EVOM™ Manual) to monitor the barrier integrity.
    Note: Washing procedure has an enormous impact on the TEER. Thus, consider to either always measure before washing or after washing to allow comparison of the results. We usually measure the TEER after the first washing step.
24. Add fresh **AEGM** (250 µl apical, 500 µl basal) and incubate at 37°C and 5% CO_2 for 2-3 days.

Day 12:

25. Wash cells twice with HBSS+ on a horizontal shaker for 5 min and measure TEER.
26. Add 500 µl **ALI medium** to the basal compartment only (from now on, cells are cultured under **"ALI conditions"**) and incubate at 37°C and 5% CO_2.
    Note: Cells should reach confluence with 3-4 days after seeding, which can be recognized by a peak in TEER and by the fact that no more medium from the basal compartment leaks through the membrane.

27. Medium should be changed every 2-3 days and ALI cultures should be washed once a week with HBSS+ to remove dead cells and mucus.
28. After 2-3 weeks under ALI conditions, cells are usually fully differentiated and can be used for infection experiments. The status of differentiation can be checked only by immunofluorescence staining of ciliated cells (*e.g.*, with anti-tubulin α) because the non-transparent membrane does not allow visualization of the cell layer by light microscopy.
    Note: Prior to infection experiments, ALI cultures should be washed and maintained in ALI medium <u>without antibiotics</u> for at least one day.
