# Supplementary material for: Primary cell culture systems to investigate host-pathogen interactions in bacterial respiratory tract infections of livestock
Source: Front Cell Infect Microbiol. 2025 May 9;15:1565513. doi: 10.3389/fcimb.2025.1565513 (PMC12098631; doi:10.3389/fcimb.2025.1565513)
Supplement: Supplementary file 3 [file SupplementaryFile3.pdf]

Supplementary Material

1 General protocol for preparation of (porcine) precision-cut lung slices (PCLS)

Provided by Désirée Schaaf and Yenehiwot B. Weldearegay (Institute for Microbiology, University of Veterinary Medicine Hannover, Germany)

1.1 Important Notes

This protocol should be considered a guideline, and specific adjustments according to user needs should be implemented, including any changes appertaining to the animal species of interest, as the following protocol is prepared using the pig as an example. Photos provided by Désirée Schaaf and Sandy Müller.

Protocol according to Vötsch *et al.*, 2020 (https://doi.org/10.1080/21505594.2020.1858604).

1.2 Preparation of Media

RPMI 1640 medium:

	**Company**	**Cat. No.**	**Final concentration**
RPMI 1640	Thermo Fisher Scientific	21875034	
Penicillin/Streptomycin	Merck	P4333	100 U, 0.1 mg/ml
Gentamicin	Carl Roth	HN09.2	50 µg/ml
Amphotericin B	Merck	A2942	2.5 µg/ml
Kanamycin	Carl Roth	T832.2	50 µg/ml
Clotrimazol	Merck	PHR1058	1 µg/ml

Agarose (1.5%):

1. Prepare two-fold RPMI 1640 medium by dissolving one vial of powder (Merck, Cat. No. R1383) in 500 ml sterile *Aqua dest.*, store at 4°C
2. Dissolve 7.5 g low melting temperature agarose (Gerbu, Cat. No. 1744; final concentration 1.5%) in 250 ml sterile *Aqua dest.* by boiling in the microwave (900 W)
3. Add 250 ml of cold two-fold RPMI 1640 medium to the agarose and keep it in a water bath at 37°C

1.3 Procedure

1. Transport lungs from freshly slaughtered pigs on ice to the laboratory
2. Separate the cranial and middle (and intermediate) lung lobes from the lung using a butcher's knife (Photo 1, encircled areas)

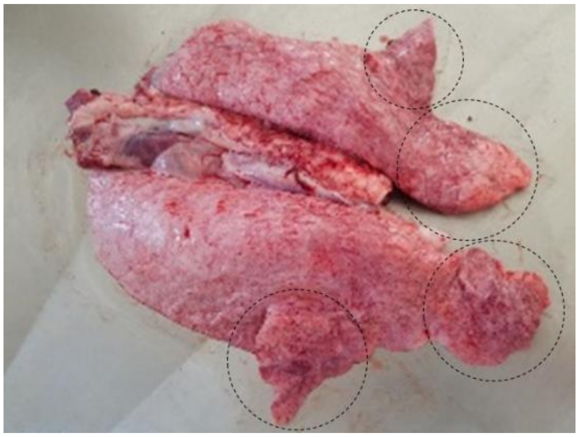
Photo 1

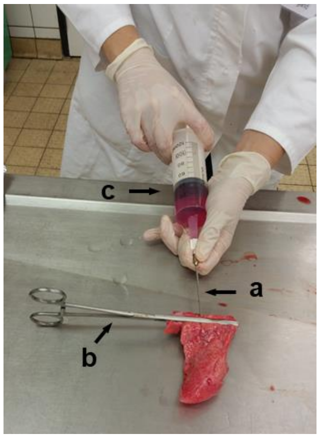

Photo 2

3. Insert a cannula (Photo 2, a) into the largest bronchus and push it <u>carefully</u> forward as far as possible without piercing the bronchus, close the lobe with a tissue clamp (Photo 2, b)
4. Place a 50 ml-syringe filled with warm agarose (Photo 2, c) on the cannula and fill the lung lobe <u>slowly</u> with agarose until it has visibly enlarged and become firm
5. Cover the filled lung lobes with ice until the agarose has completely solidified
6. Stamp out cylindrical portions of the lung lobe (with a bronchiole in the center) using a tissue coring tool (diameter 8-10 mm; Photo 3), keep on ice until slicing

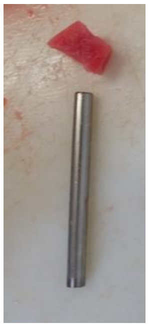
Photo 3

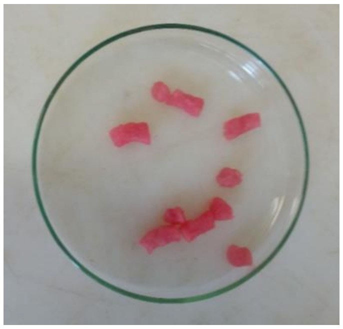
Photo 4

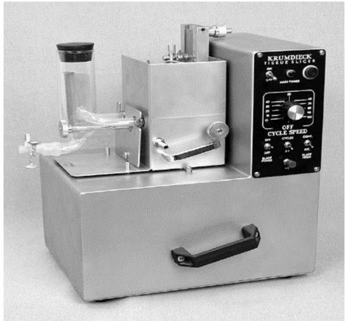
Photo 5

7. Place cylindrical portions (Photo 4) in a Krumdieck tissue slicer (model MD 4000-01, TSE Systems; Photo 5) filled with 500 ml RPMI 1640 medium (Thermo Fisher Scientific, Cat. No. 21875034) and cut slices with a thickness of about 300 µm
8. Collect slices in a 100 ml-glass bottle (lid with a hole) filled with pre-warmed (37°C) RPMI 1640 medium supplemented with antibiotics and antimycotics (see above) and bubble with a normoxic gas mixture at 37°C for approximately two hours to remove agarose from the airways (Photo 6; modified from Paddenberg *et al.*, 2014; doi:10.3791/50970)

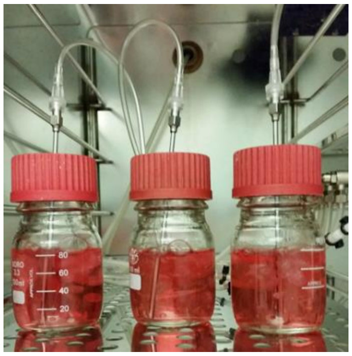

Photo 6

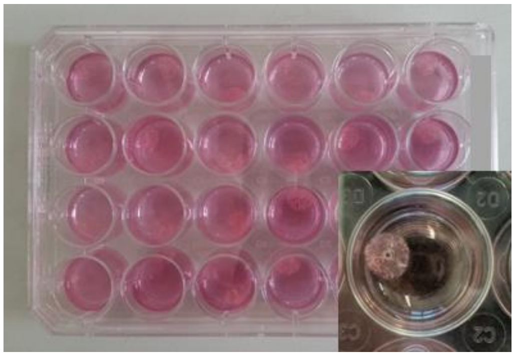

Photo 7

9. Transfer slices to a 24-well plate filled with pre-warmed (37°C) RPMI 1640 medium supplemented with antibiotics and antimycotics (1 ml/well) and incubate at 37°C and 5% CO_2 for 1-2 days (Photo 7, enlarged view of a slice in the inlay)
10. Check ciliary activity using a light microscope (Leica DMi1; Leica) and select slices with at least 80% ciliary activity (Photo 8, cilia indicated by arrow)

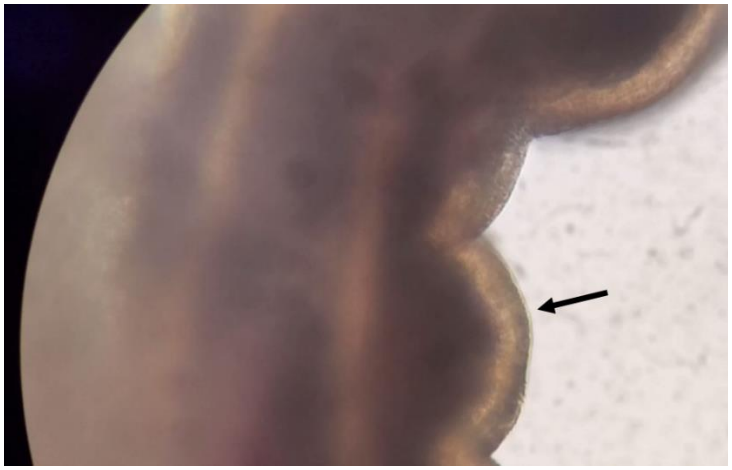

Photo 8

11. Prior to infection experiments slices have to be washed with phosphate-buffered saline (PBS) and incubated for 1-2 days in RPMI 1640 medium <u>without</u> antibiotics/antimycotics
