# Supplementary material for: Primary cell culture systems to investigate host-pathogen interactions in bacterial respiratory tract infections of livestock
Source: Front Cell Infect Microbiol. 2025 May 9;15:1565513. doi: 10.3389/fcimb.2025.1565513 (PMC12098631; doi:10.3389/fcimb.2025.1565513)
Supplement: Supplementary file 4 [file Table1.pdf]

Supplementary Tables

Species	Reference	Medium	Protease	DNase	DTT	Incubation
Bovine	Goris *et al.*, 2009; according to Bals *et al.*, 2004	MEM	0.1%	10 µg/ml	0.5 mg/ml	24 h, 4°C
	Cozens *et al.*, 2018a	DMEM + Ham's F12	1 mg/ml	10 µg/ml	1 mg/ml	Overnight, 4°C
Ovine	Radi and Ackermann, 2004	MEM	0.15%	0.01%	-	24-48 h, 4°C
	Mao *et al.*, 2009	M199	0.6 mg/ml	-	-	Overnight, 4°C
	Xue *et al.*, 2015	DMEM	1.5 mg/ml	10 µg/ml	-	48 h, 4°C
	O'Boyle *et al.*, 2017	DMEM + Ham's F12	1 mg/ml	10 µg/ml	1 mg/ml	Overnight, 4°C
Caprine	Strassle *et al.*, 2021; according to Gultom *et al.*, 2020	MEM (+ 15 mM HEPES)	0.1%	0.001%	-	48 h, 4°C
Equine	Schwab *et al.*, 2010	DMEM	1%	0.1%	-	Overnight, 4°C
	Oslund *et al.*, 2010	MEM	0.2%	-	-	Overnight, 4°C
	Abraham *et al.*, 2011	HBSS (Ca^{2+}/Mg^{2+} free)	0.25% trypsin-0.6 mM EDTA			2 h, 37°C
	Quintana *et al.*, 2011	MEM	1.4%	0.1%	-	48 h, 4°C (?)
Porcine	Mao *et al.*, 2009	M199	0.6 mg/ml	-	-	Overnight, 4°C
	Khoufache *et al.*, 2010	DMEM + Ham's F12	0.1%	-	-	Overnight, 4°C
	Lam *et al.*, 2011	DMEM	1.4 mg/ml	0.1 mg/ml	-	72 h, 4°C
	Bateman *et al.*, 2013; according to Busch *et al.*, 2008	MEM (Ca^{2+}/Mg^{2+} free)	1.4 mg/ml	0.1 mg/ml	-	72 h, 4°C
	Meng *et al.*, 2016	EMEM	1 mg/ml	0.1 mg/ml	-	48 h, 4°C
	Wang *et al.*, 2018	MEM	1 mg/ml	0.1 mg/ml	-	24-48 h, 2-8°C

Supplementary table 1. Comparison of different "digestion buffers". Antibiotics/antimycotics are not listed. DTT: dithiothreitol; MEM: Minimum Essential Medium; DMEM: Dulbecco's Modified Eagle's Medium; HBSS: Hank's Balanced Salt Solution; EDTA: Ethylenediaminetetraacetic acid; "-": no information available or not included.

**Species**	**Reference**	**Medium**	**Serum**	**BPE**	**EGF**	**Insulin**	**Cortisone**	**Epinephrin**	**T3**	**Transferrin**	**RA**	**Other factors**
Bovine	Goris *et al.*, 2009	AEGM	FBS (10%)	0.4%	10 ng/ml	5 µg/ml	0.5 µg/ml	0.5 µg/ml	6.7 ng/ml	10 µg/ml	0.1 ng/ml	-
	Cozens *et al.*, 2018a	DMEM + Ham's F12	FBS (10%)	-	-	-	-	-	-	-	-	-
Ovine	Mao *et al.*, 2009	BEGM	FBS (5%)	60 µg/ml	0.5 ng/ml	5 µg/ml	0.5 µg/ml	0.5 µg/ml	6.5 ng/ml	10 µg/ml	0.1 ng/ml	Cholera toxin (10 ng/ml)
	Xue *et al.*, 2015	BEGM	FBS (5%)	0.4%	0.1%	0.1%	0.1%	0.1%	0.1%	0.1%	0.1%	-
	O'Boyle *et al.*, 2017	AEGM	-	4 µl/ml	10 ng/ml	5 µg/ml	0.5 µg/ml	0.5 µg/ml	6.7 ng/ml	10 µg/ml	0.1 ng/ml	-
Caprine	Strassle *et al.*, 2021; according to Gultom *et al.*, 2020	LHC basal medium	BSA (0.5 mg/ml)	14 µg/ml	25 ng/ml	5 µg/ml	0.48 µg/ml	0.6 µg/ml	6.7 ng/ml	10 µg/ml	50 nM (EC23)	HEPES (0.015 mM/l)<br>PE (70 µg/ml)<br>Trace elements<br>A83-01 (1 µM/l)<br>Y27632 (5 µM/l)<br>Isoprotenerol (3 µM/l)
Equine	Oslund *et al.*, 2010	DMEM + Ham's F12	BSA (0.5 mg/ml)	15 µg/ml	5 ng/ml	4 µg/ml	0.1 µM	-	-	-	-	Cholera toxin (20 ng/ml)
	Abraham *et al.*, 2011	AEGM	FBS (10%)	0.4%	10 ng/ml	5 µg/ml	0.5 µg/ml	0.5 µg/ml	6.7 ng/ml	10 µg/ml	0.1 ng/ml	-
Porcine	Mao *et al.*, 2009	BEGM	FBS (5%)	60 µg/ml	0.5 ng/ml	5 µg/ml	0.5 µg/ml	0.5 µg/ml	6.5 ng/ml	10 µg/ml	0.1 ng/ml	Cholera toxin (10 ng/ml)
	Lam *et al.*, 2011	DMEM + AEBM	BSA (1.5 ng/ml)	52 µg/ml	0.5 ng/ml	5 µg/ml	0.5 µg/ml	0.5 µg/ml	-	10 µg/ml	15 ng/ml	-
	Bateman *et al.*, 2013	BEGM	-	0.4%	0.1%	0.1%	0.1%	0.1%	0.1%	0.1%	0.1%	-
	Meng *et al.*, 2016	BEBM	BSA (0.5 mg/ml)	14 µg/ml	50 ng/ml	5 µg/ml	72 ng/ml	0.6 µg/ml	6.7 ng/ml	20 µg/ml	15 ng/ml	-
	Wang *et al.*, 2018	BEGM	-	0.4%	0.1%	0.1%	0.1%	0.1%	0.1%	0.1%	0.1%	-

Supplementary table 2. Comparison of different "submerged growth media". Antibiotics/antimycotics are not listed. BPE: Bovine pituitary extract; EGF: Epidermal growth factor; T3: Triiodo-L-thyronine; RA: Retinoic acid; AEGM: Airway Epithelial Cell Growth Medium (Promocell); DMEM: Dulbecco's Modified Eagle's Medium; BEGM: Bronchial Epithelial Cell Growth Medium (Lonza); FBS: Fetal bovine serum; BSA: Bovine serum albumin; AEBM: Airway Epithelial Cell Basal Medium (Promocell); BEBM: Bronchial Epithelial Cell Basal Medium (Lonza) ; PE: Phosphorylethanolamine; "-": no information available or not included.

Species	Reference	Company	Material	Diameter	Area	Pore size	Pore density	Cells/insert	Cells/cm²	Coating
Bovine	Goris *et al.*, 2009	Greiner	PET	6.5 mm	0.3 cm²	0.4 µm	?	2.5×10^5	8×10^5	Collagen type I
	Cozens *et al.*, 2018a	Greiner	PET	12 mm	1.1 cm²	0.4 µm	1×10^8/cm²	2.5×10^5	2.3×10^5	-
Caprine	Strassle *et al.*, 2021	Corning	PET	6.5 mm	0.3 cm²	0.4 µm	4×10^6/cm²	2×10^5	~ 6×10^5	Collagen type IV
Ovine	Radi and Ackermann, 2004	Millipore	PC	12 mm	0.6 cm²	0.4 µm	-	2.4×10^5	4×10^5	Collagen type IV
	Mao *et al.*, 2009	Corning	PTFE	6.5 mm	0.3 cm²	0.4 µm	-	5×10^4	1.7×10^5	Collagen type I
	Xue *et al.*, 2015	Millipore	PC	12 mm	0.6 cm²	0.4 µm	-	1×10^5	1.7×10^5	Collagen type I
	O'Boyle *et al.*, 2017	Greiner	PET	12 mm	1.1 cm²	0.4 µm	1×10^8/cm²	2.5×10^5	2.3×10^5	-
Equine	Schwab *et al.*, 2010	Corning	PET	24 mm	4.7 cm²	0.4 µm	4×10^6/cm²	5×10^6	1.1×10^6	Collagen type IV
	Oslund *et al.*, 2010	Corning	PC	12 mm	1.1 cm²	0.4 µm	1×10^8/cm²	5×10^4	4.5×10^4	-
	Abraham *et al.*, 2011	Greiner	PET	6.5 mm	0.3 cm²	0.4 µm	?	3.6×10^5	~ 1.2×10^6	Collagen type I
	Quintana *et al.*, 2011	Corning	-	-	-	-	-	-	-	Collagen type IV
Porcine	Mao *et al.*, 2009	Corning	PTFE	6.5 mm	0.3 cm²	0.4 µm	-	5×10^4	1.7×10^5	Collagen type I
	Lam *et al.*, 2011	Corning	PET	6.5 mm	0.3 cm²	0.4 µm	4×10^6/cm²	2×10^5	6.6×10^5	Collagen type I
	Bateman *et al.*, 2013	Corning	PET	12 mm	1.1 cm²	0.4 µm	4×10^6/cm²	1.2×10^5	1×10^5	Collagen type VI
	Meng *et al.*, 2016	Corning	PC	6.5 mm	0.3 cm²	0.4 µm	1×10^8/cm²	2.5×10^5	8×10^5	Collagen type IV
	Wang *et al.*, 2018	Corning	PET	6.5 mm	0.3 cm²	0.4 µm	4×10^6/cm²	8×10^5	2.6×10^6	Collagen

Supplementary table 3. Comparison of different porous membrane supports. PET: Polyethylene terephthalate (polyester); PC: Polycarbonate; PTFE: Polytetrafluoroethylene; “-“: no information available.

Species	Reference	Medium	Serum	BPE	EGF	Insulin	Cortisone	Epi	T3	Transferrin	RA	Other factors
Bovine	Goris *et al.*, 2009	DMEM + Ham's F12	Ultroser G (2%)	-	-	-	-	-	-	-	15 ng/ml	-
	Cozens *et al.*, 2018a	DMEM + AEBM	-	0.4%	10 ng/ml	5 µg/ml	0.5 µg/ml	0.5 µg/ml	6.7 ng/ml	10 µg/ml	100 nM	-
Caprine	Strassle *et al.*, 2021; according to Gultom *et al.*, 2020	DMEM + LHC basal medium	BSA (0.5 mg/ml)	14 µg/ml	5.5 ng/ml	5 µg/ml	0.48 µg/ml	0.6 µg/ml	6.7 ng/ml	10 µg/ml	50 nM (EC23)	DAPT (5 µM/l)
Ovine	Radi and Ackermann, 2004	DMEM + Ham's F12	Ultroser G (2%)	-	-	-	-	-	-	-	-	-
	Mao *et al.*, 2009	BEGM	-	60 µg/ml	0.5 ng/ml	5 µg/ml	0.5 µg/ml	0.5 µg/ml	6.5 ng/ml	10 µg/ml	100 nM	Cholera toxin (10 ng/ml)
	Xue *et al.*, 2015	-	Ultroser G (2%)	-	-	-	-	-	-	-	-	-
	O'Boyle *et al.*, 2017	DMEM + AEBM	-	0.4%	10 ng/ml	5 µg/ml	0.5 µg/ml	0.5 µg/ml	6.7 ng/ml	10 µg/ml	100 nM	-
Equine	Oslund *et al.*, 2010	DMEM + Ham's F12	BSA (0.5 mg/ml)	15 µg/ml	5 ng/ml	4 µg/ml	0.1 µM	-	-	5 µg/ml	-	Cholera toxin (20 ng/ml)
	Abraham *et al.*, 2011	DMEM + Ham's F12	Ultroser G (2%)	-	-	-	-	-	-	-	15 ng/ml	-
	Quintana *et al.*, 2011	DMEM + Ham's F12	Ultroser G (2%)	-	-	-	-	-	-	-	-	-
Porcine	Mao *et al.*, 2009	BEGM	-	60 µg/ml	0.5 ng/ml	5 µg/ml	0.5 µg/ml	0.5 µg/ml	6.5 ng/ml	10 µg/ml	100 nM	Cholera toxin (10 ng/ml)
	Lam *et al.*, 2011	DMEM + AEBM	BSA (1.5 ng/ml?)	52 µg/ml	0.5 ng/ml	5 µg/ml	0.5 µg/ml	0.5 µg/ml	-	10 µg/ml	15 ng/ml	-
	Bateman *et al.*, 2013	DMEM + BEBM	BSA (1%)	1%	1.5 ng/ml	50 µg/ml	1.4 µM	2.7 µM	9.7nM	10 ng/ml	100 nM	-
	Meng *et al.*, 2016	DMEM + BEGM	BSA (0.5 mg/ml)	14 µg/ml	1 ng/ml	5 µg/ml	72 ng/ml	0.6 µg/ml	6.7 ng/ml	20 µg/ml	15 ng/ml	-
	Wang *et al.*, 2018	BEGM	Ultroser G (2%)	-	-	-	-	-	-	-	15 ng/ml	-

Supplementary table 4. Comparison of different "ALI media". Antibiotics/antimycotics are not listed. BPE: Bovine pituitary extract; EGF: Epidermal growth factor; Epi: Epinephrine; T3: Triiodo-L-thyronine; RA: Retinoic acid; DMEM: Dulbecco's Modified Eagle's Medium; AEBM: Airway Epithelial Cell Basal Medium (Promocell); BEGM: Bronchial Epithelial Cell Growth Medium (Lonza); BSA: Bovine serum albumin; BEBM: Bronchial Epithelial Cell Basal Medium (Lonza); "-": no information available or not included.

**Structure**	**Antigen**	**Reference**
Tight junctions/adherens junctions	ZO-1	Abraham *et al.*, 2011; Xue *et al.*, 2015; Ma *et al.*, 2016; O'Boyle *et al.*, 2017; Cozens *et al.*, 2018b; Wang *et al.*, 2018; Wang *et al.*, 2020; Strassle *et al.*, 2021; Genna *et al.*, 2023
	β-catenin	Wu *et al.*, 2016
	Occludin	Lam *et al.*, 2011; Meng *et al.*, 2016
Cilia	β-tubulin	Lam *et al.*, 2011; Meng *et al.*, 2016; O'Boyle *et al.*, 2017; Cozens *et al.*, 2018b; Su *et al.*, 2020; Wang *et al.*, 2020; Strassle *et al.*, 2021; Qin *et al.*, 2023
	Acetylated α-tubulin	Genna *et al.*, 2023
	Tubulin IV	Xue *et al.*, 2015; Ma *et al.*, 2016
Mucus/goblet cells	MUC5AC	Xue *et al.*, 2015; Ma *et al.*, 2016; Meng *et al.*, 2016; Wu *et al.*, 2016; Cozens *et al.*, 2018b; Genna *et al.*, 2023
	MUC5B	Wang *et al.*, 2018; Wang *et al.*, 2020
	Jacalin	O'Boyle *et al.*, 2017
Basal cells	P63	O'Boyle *et al.*, 2017; Cozens *et al.*, 2018b
	Cytokeratin	Abraham *et al.*, 2011; Xue *et al.*, 2015; Ma *et al.*, 2016; Wu *et al.*, 2016; Genna *et al.*, 2023

Supplementary table 5. Antibodies used for the visualization of specific structures in ALI cultures by immunofluorescence staining or immunohistochemistry.

References

Abraham, G., Zizzadoro, C., Kacza, J., Ellenberger, C., Abs, V., Franke, J., et al. (2011). Growth and differentiation of primary and passaged equine bronchial epithelial cells under conventional and air-liquid-interface culture conditions. *BMC Vet Res* 7**,** 26. doi: 10.1186/1746-6148-7-26.

Bals, R., Beisswenger, C., Blouquit, S., and Chinet, T. (2004). Isolation and air-liquid interface culture of human large airway and bronchiolar epithelial cells. *J Cyst Fibros* 3 Suppl 2**,** 49-51. doi: 10.1016/j.jcf.2004.05.010.

Bateman, A.C., Karasin, A.I., and Olsen, C.W. (2013). Differentiated swine airway epithelial cell cultures for the investigation of influenza A virus infection and replication. *Influenza Other Respir Viruses* 7(2)**,** 139-150. doi: 10.1111/j.1750-2659.2012.00371.x.

Busch, M.G., Bateman, A.C., Landolt, G.A., Karasin, A.I., Brockman-Schneider, R.A., Gern, J.E., et al. (2008). Identification of amino acids in the HA of H3 influenza viruses that determine infectivity levels in primary swine respiratory epithelial cells. *Virus Res* 133(2)**,** 269-279. doi: 10.1016/j.virusres.2008.01.014.

Cozens, D., Grahame, E., Sutherland, E., Taylor, G., Berry, C.C., and Davies, R.L. (2018a). Development and optimization of a differentiated airway epithelial cell model of the bovine respiratory tract. *Sci Rep* 8(1)**,** 853. doi: 10.1038/s41598-017-19079-y.

Cozens, D., Sutherland, E., Marchesi, F., Taylor, G., Berry, C.C., and Davies, R.L. (2018b). Temporal differentiation of bovine airway epithelial cells grown at an air-liquid interface. *Sci Rep* 8(1)**,** 14893. doi: 10.1038/s41598-018-33180-w.

Genna, V.G., Adamo, D., Galaverni, G., Lepore, F., Boraldi, F., Quaglino, D., et al. (2023). Validation of airway porcine epithelial cells as an alternative to human in vitro preclinical studies. *Sci Rep* 13(1)**,** 16290. doi: 10.1038/s41598-023-43284-7.

Goris, K., Uhlenbruck, S., Schwegmann-Wessels, C., Kohl, W., Niedorf, F., Stern, M., et al. (2009). Differential sensitivity of differentiated epithelial cells to respiratory viruses reveals different viral strategies of host infection. *J Virol* 83(4)**,** 1962-1968. doi: 10.1128/JVI.01271-08.

Gultom, M., Laloli, L., and Dijkman, R. (2020). Well-Differentiated Primary Mammalian Airway Epithelial Cell Cultures. *Methods Mol Biol* 2203**,** 119-134. doi: 10.1007/978-1-0716-0900-2_10.

Khoufache, K., Cabaret, O., Farrugia, C., Rivollet, D., Alliot, A., Allaire, E., et al. (2010). Primary in vitro culture of porcine tracheal epithelial cells in an air-liquid interface as a model to study airway epithelium and Aspergillus fumigatus interactions. *Med Mycol* 48(8)**,** 1049-1055. doi: 10.3109/13693786.2010.496119.

Lam, E., Ramke, M., Groos, S., Warnecke, G., and Heim, A. (2011). A differentiated porcine bronchial epithelial cell culture model for studying human adenovirus tropism and virulence. *J Virol Methods* 178(1-2)**,** 117-123. doi: 10.1016/j.jviromet.2011.08.025.

Ma, Y., Han, F., Liang, J., Yang, J., Shi, J., Xue, J., et al. (2016). A species-specific activation of Toll-like receptor signaling in bovine and sheep bronchial epithelial cells triggered by Mycobacterial infections. *Mol Immunol* 71**,** 23-33. doi: 10.1016/j.molimm.2016.01.004.

Mao, H., Wang, Y., Yuan, W., and Wong, L.B. (2009). Ciliogenesis in cryopreserved mammalian tracheal epithelial cells cultured at the air-liquid interface. *Cryobiology* 59(3)**,** 250-257. doi: 10.1016/j.cryobiol.2009.07.012.

Meng, F., Wu, N.H., Seitz, M., Herrler, G., and Valentin-Weigand, P. (2016). Efficient suilysin-mediated invasion and apoptosis in porcine respiratory epithelial cells after streptococcal infection under air-liquid interface conditions. *Sci Rep* 6**,** 26748. doi: 10.1038/srep26748.

O'Boyle, N., Sutherland, E., Berry, C.C., and Davies, R.L. (2017). Temporal dynamics of ovine airway epithelial cell differentiation at an air-liquid interface. *PLoS One* 12(7)**,** e0181583. doi: 10.1371/journal.pone.0181583.

Oslund, K.L., Adamson, G., and Wu, R. (2010). Evaluation of MUC5AC expression and upregulation in airway epithelial cells of horses. *Am J Vet Res* 71(6)**,** 690-696. doi: 10.2460/ajvr.71.6.690.

Qin, L., Meng, F., He, H., Li, S., Zhang, H., Sun, Y., et al. (2023). Inflammation plays a critical role in damage to the bronchiolar epithelium induced by Trueperella pyogenes in vitro and in vivo. *Infect Immun* 91(12)**,** e0027323. doi: 10.1128/iai.00273-23.

Quintana, A.M., Landolt, G.A., Annis, K.M., and Hussey, G.S. (2011). Immunological characterization of the equine airway epithelium and of a primary equine airway epithelial cell culture model. *Vet Immunol Immunopathol* 140(3-4)**,** 226-236. doi: 10.1016/j.vetimm.2010.12.008.

Radi, Z.A., and Ackermann, M.R. (2004). Growth of differentiated ovine tracheal epithelial cells in vitro. *J Vet Med A Physiol Pathol Clin Med* 51(4)**,** 167-170. doi: 10.1111/j.1439-0442.2004.00620.x.

Schwab, U.E., Fulcher, M.L., Randell, S.H., Flaminio, M.J., and Russell, D.G. (2010). Equine bronchial epithelial cells differentiate into ciliated and mucus producing cells in vitro. *In Vitro Cell Dev Biol Anim* 46(2)**,** 102-106. doi: 10.1007/s11626-009-9258-6.

Strassle, M., Laloli, L., Gultom, M., V'Kovski, P., Stoffel, M.H., Crespo Pomar, S., et al. (2021). Establishment of caprine airway epithelial cells grown in an air-liquid interface system to study caprine respiratory viruses and bacteria. *Vet Microbiol* 257**,** 109067. doi: 10.1016/j.vetmic.2021.109067.

Su, A., Tong, J., Fu, Y., Muller, S., Weldearegay, Y.B., Becher, P., et al. (2020). Infection of bovine well-differentiated airway epithelial cells by Pasteurella multocida: actions and counteractions in the bacteria-host interactions. *Vet Res* 51(1)**,** 140. doi: 10.1186/s13567-020-00861-2.

Wang, H., He, L., Liu, B., Feng, Y., Zhou, H., Zhang, Z., et al. (2018). Establishment and comparison of air-liquid interface culture systems for primary and immortalized swine tracheal epithelial cells. *BMC Cell Biol* 19(1)**,** 10. doi: 10.1186/s12860-018-0162-3.

Wang, H., Zhang, Z., Xie, X., Liu, B., Wei, Y., Gan, Y., et al. (2020). Paracellular Pathway-Mediated Mycoplasma hyopneumoniae Migration across Porcine Airway Epithelial Barrier under Air-Liquid Interface Conditions. *Infect Immun* 88(10). doi: 10.1128/IAI.00470-20.

Wu, N.H., Yang, W., Beineke, A., Dijkman, R., Matrosovich, M., Baumgartner, W., et al. (2016). The differentiated airway epithelium infected by influenza viruses maintains the barrier function despite a dramatic loss of ciliated cells. *Sci Rep* 6**,** 39668. doi: 10.1038/srep39668.

Xue, D., Ma, Y., Li, M., Li, Y., Luo, H., Liu, X., and Wang, Y. (2015). Mycoplasma ovipneumoniae induces inflammatory response in sheep airway epithelial cells via a MyD88-dependent TLR signaling pathway. *Vet Immunol Immunopathol* 163(1-2)**,** 57-66. doi: 10.1016/j.vetimm.2014.11.008.
